# A Set of Novel Venom Proteins Enables Parasitoid Wasps to Exploit Older Hosts and Coexist with Competitors

**DOI:** 10.1002/advs.202512654

**Published:** 2025-11-05

**Authors:** Junwei Zhang, Zhi Dong, Yifeng Sheng, Jieyu Shan, Ting Feng, Wenqi Shi, Zixuan Xu, Zeying Wang, Qichao Zhang, Ying Wang, Jianhua Huang, Jiani Chen

**Affiliations:** ^1^ Zhejiang Key Laboratory of Biology and Ecological Regulation of Crop Pathogens and Insects Institute of Insect Sciences College of Agriculture and Biotechnology Zhejiang University Hangzhou 310058 China; ^2^ Ministry of Agriculture Key Laboratory of Molecular Biology of Crop Pathogens and Insects Zhejiang University Hangzhou 310058 China

**Keywords:** gene duplication, interspecific competition, parasitoid wasps, species coexistence, venom protein genes

## Abstract

Interspecific competition can drive species coexistence through niche differentiation, yet the underlying mechanisms remain unclear. Parasitoid wasps are a group of parasitic insects that rely on host nutrients to complete their development, exhibiting intense interspecific competition. Here, two parasitoid wasps, *Asobara japonica* and *Leptopilina drosophilae* are employed, which share the common host *Drosophila melanogaster*, as a model system to investigate the mechanisms governing species coexistence. *A. japonica* employs venom‐induced host manipulation to exploit older hosts is found, thereby avoiding competition and coexisting with its competitor, *L. drosophilae*. Through integrated multi‐omics and functional studies, a set of DUF4803‐domain venom proteins is identified that induce the apoptosis‐mediated degradation of host imaginal discs. This process elevates *dilp8* expression, causing a delay in host development that is essential for the successful development of *A. japonica* offspring within older hosts. How gene duplication and the subsequent functional specialization of these DUF4803‐domain genes facilitated this mechanism, allowing host resource partitioning through temporal niche differentiation, is further revealed. The study suggests that this adaptive strategy minimizes evolutionary trade‐offs and advances the understanding of species coexistence mechanisms.

## Graphical abstract

This study elucidates a molecular mechanism enabling species coexistence between parasitoid wasps, showing how *Asobara japonica* uses a set of novel venom proteins to delay host development and exploit older hosts, thereby achieving temporal niche partitioning with *Leptopilina drosophilae*.

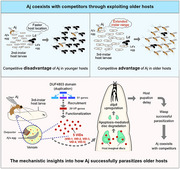

## 1 Introduction

Interspecific competition is a fundamental ecological process that shapes biological communities by influencing species distributions, abundance, and evolutionary trajectories, thereby maintaining ecosystem stability and driving adaptive evolution.^[^ ^1^, ^2^, ^3^, ^4^, ^5^, ^6^ ^]^ The theoretical foundation of interspecific competition was initially established by the competitive exclusion principle, which posited that two species competing for identical resources cannot coexist indefinitely, inevitably leading to the exclusion of the weaker competitor.^[^ ^7^, ^8^, ^9^, ^10^, ^11^ ^]^ This principle, rooted in Darwin's theory of natural selection and Malthusian population dynamics, has profoundly influenced ecological thinking.^[^ ^1^, ^12^ ^]^ However, as empirical evidence accumulated, it has become clear that coexistence, rather than exclusion, represents a more common outcome of interspecific competition in natural systems.^[^ ^13^, ^14^, ^15^, ^16^, ^17^, ^18^, ^19^ ^]^ This shift reflects an evolving understanding of how species adapt to competitive pressures, leading to the development of the competitive niche shift principle.^[^ ^1^, ^15^, ^20^ ^]^ This principle emphasizes that species can coexist through strategies such as resource partitioning, temporal or spatial segregation, and adaptive trait divergence, which reduce direct competition and promote coexistence. For example, Darwin's finches on the Galápagos Islands exhibit adaptive trait divergence, evolving distinct beak morphologies to exploit different seed types as food to avoid competition.^[^ ^2^, ^21^, ^22^ ^]^ In African savannas, zebras, wildebeests, and gazelles coexist through the partitioning of grazing resources, driven by seasonal migration.^[^ ^23^ ^]^ Despite the extensive cases of coexistence in nature, the mechanisms enabling species to coexist under competitive pressures remain poorly understood.

Parasitoid wasps (also known as parasitoids) represent one of the most diverse groups of insects, with an estimated 150 000–600 000 species that rely on parasitizing other insects to complete their life cycles.^[^ ^24^, ^25^ ^]^ Their ecological ubiquity is remarkable, as nearly every insect species serves as a host for one or more parasitoid species.^[^ ^26^ ^]^ To ensure successful parasitism and offspring survival, parasitoid wasps have evolved sophisticated biochemical arsenals, including venom, polydnaviruses (PDVs), teratocytes, ovarian fluid, and larval secretions.^[^ ^27^, ^28^, ^29^, ^30^, ^31^ ^]^ Among them, venom proteins are particularly notable for their rapid evolutionary adaptation, enabling precise manipulation of host physiology and immune responses to create an optimal environment for parasitoid offspring development.^[^ ^32^, ^33^ ^]^ However, the development of parasitoid offspring is entirely dependent on the finite resources of a single host, leading to intense interspecific competition when multiple parasitoid species target the same host.^[^ ^34^, ^35^ ^]^ This competition, driven by resource limitations, directly impacts parasitoid survival and fitness. Recent studies suggest that several adaptations observed in parasitoid wasps are likely evolutionary responses to mitigate interspecific competition and optimize resource utilization. For example, parasitoid wasps exhibit adaptability in targeting different host ranges—from specialists that parasitize a single host species to generalists that exploit multiple hosts.^[^ ^32^, ^36^, ^37^ ^]^ These adaptations highlight the diverse strategies that parasitoids use to reduce interspecific competition and increase parasitism success. Nevertheless, despite intense interspecific competition for shared host resources, multiple parasitoid species often coexist in the same habitat. This ability to coexist under competitive pressures makes parasitoid wasps an ideal model system for studying the mechanisms of species coexistence.

In 2018, we conducted field surveys in *Myrica rubra* orchards in Taizhou (28°50′N, 120°34′E), Zhejiang Province, China, to identify natural parasitoids of *Drosophila*. Using fruit baits, we successfully collected two parasitoid species, *Asobara japonica* (Aj) (Hymenoptera: Braconidae) and *Leptopilina drosophilae* (Ld) (Hymenoptera: Figitidae), which coexist in the same habitat and both parasitize *D. melanogaster* host larvae.^[^ ^38^, ^39^ ^]^ Given their coexistence, we used Aj and Ld to elucidate the molecular mechanisms enabling their coexistence under interspecific competition. We found that Aj avoids direct competition with Ld by exploiting older host larvae, which Ld parasitizes inefficiently, whereas Ld dominates in competition for younger hosts because of its superior host‐searching efficacy. The ability of Aj to parasitize older hosts is mediated by its venom, which induces host pupation delay, thereby providing sufficient time for parasitoid offspring to complete their development. Mechanistically, the venom of Aj triggers apoptosis in host imaginal discs, leading to elevated levels of *dilp8*, a key regulator of developmental timing, which ultimately delays host development. Further analysis revealed a set of novel venom proteins containing a conserved domain of unknown function (DUF4803), which underpins the successful exploitation of the older hosts by inducing imaginal disc apoptosis, thereby securing the competitive advantage that allows Aj to coexist with Ld through the partitioning of host resources.

## 2 Results

### 2.1 Aj shows a Competitive Disadvantage against Ld in Younger Hosts

**Figure 1:**
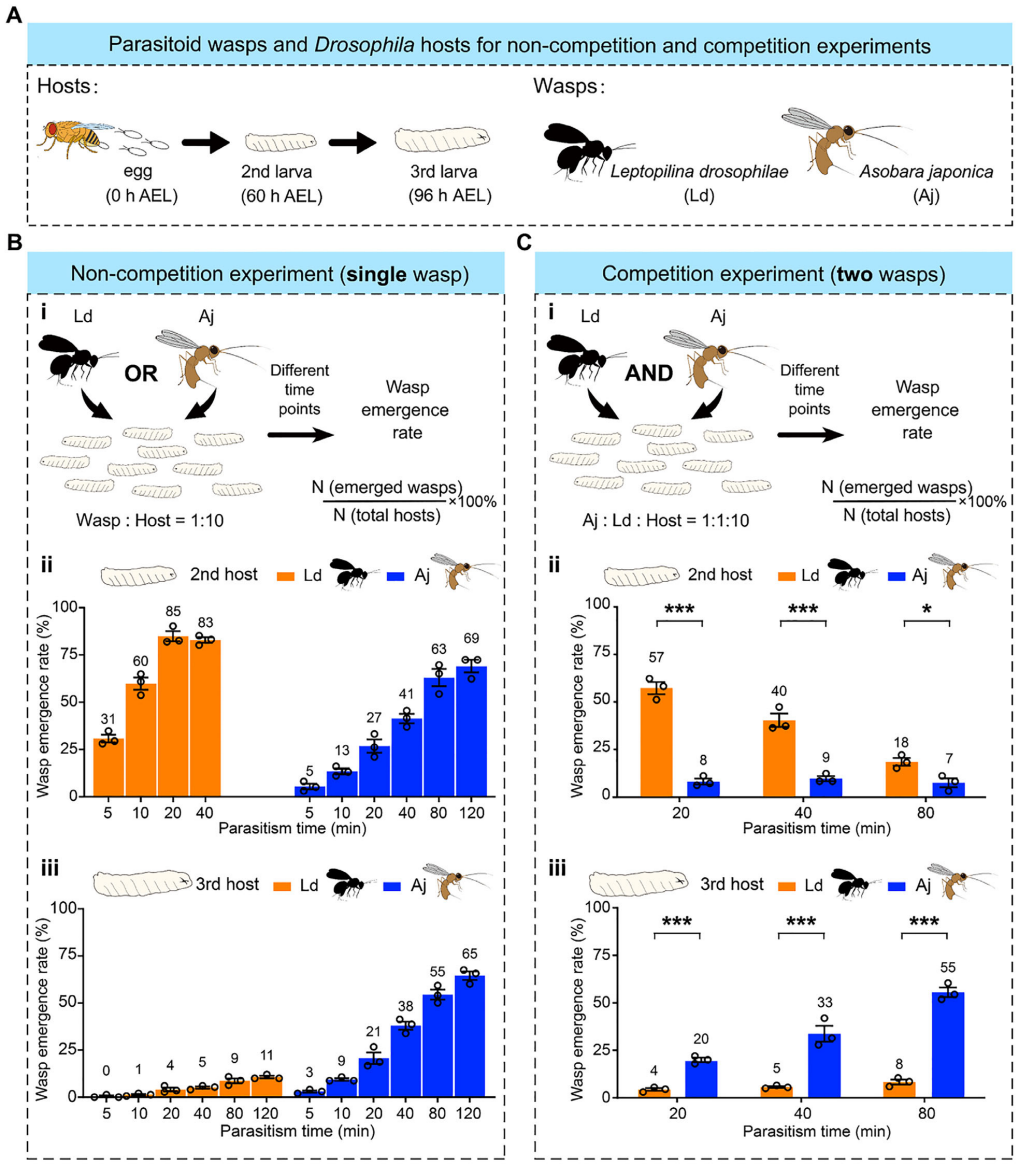
Aj coexists with Ld through parasitizing older *Drosophila* hosts. A) Experimental animals include *D. melanogaster* hosts: younger hosts (2nd‐instar larvae at 60 h AEL), older hosts (3rd‐instar larvae at 96 h AEL), and two parasitoid wasps: *A. japonica* (Aj) and *L. drosophilae* (Ld). B) Non‐competition parasitization experiments using a single parasitoid species. (B i) Schematic diagram of the non‐competition experiment. (B ii, iii) Wasp emergence rates of Aj or Ld when parasitizing 2nd‐instar hosts (ii) and 3rd‐instar hosts (iii) in non‐competition experiments at a wasp:host ratio of 1:10 at different time points. Data are presented as the mean ± SEM, with the average wasp emergence rate at each time point is shown above each bar. Three biological replicates were performed for each time point. C) Competition parasitization experiments using two parasitoid species. (C i) Schematic diagram of the competition experiment. (C ii, iii) Wasp emergence rates of Aj or Ld when parasitizing 2nd‐instar hosts (ii) and 3rd‐instar hosts (iii) in competition experiments at the Aj:Ld:host ratio of 1:1:10 at 20, 40, and 80 min. Data are presented as the mean ± SEM, with the average wasp emergence rate at each time point shown above each bar. Three biological replicates were performed for each time point. Significance was analysed by two‐way ANOVA with Sidak's multiple comparisons test (^*^, *p* < 0.05; ^***^, *p* < 0.001).

To evaluate the parasitism efficacy of Aj and Ld, we conducted a non‐competition experiment in which second‐instar *D. melanogaster* host larvae were exposed to either Aj or Ld at a 1:10 parasitoid‐to‐host ratio for different time intervals (**Figure** **1**A,B i). The emergence rates of parasitoid wasps were then quantified to assess their parasitism success (Figure 1B ii). We found that both Aj and Ld were capable of successfully completing oviposition within host larvae (Movies S1 and S2, Supporting Information). The wasp emergence rates of Ld were 31% and 60% at 5 and 10 min intervals, respectively, and exceeded 80% after 20 min (Figure 1B ii). In contrast, the wasp emergence rates of Aj increased at a slower rate, reaching 27% at the 20 min interval and exceeding 60% only after 80 min (Figure 1B ii). Behavioural observations further revealed that Ld located hosts significantly faster than Aj, requiring an average of 20.89 ± 1.69 s compared with 68.67 ± 5.91 s for Aj (Figure S1, Supporting Information). This rapid host‐searching ability likely constitutes a primary reason for the higher emergence rates of Ld over shorter time intervals, although other explanations, such as a differential ability to counteract host defenses at this developmental stage, cannot be excluded. Together, these results indicate that while both Aj and Ld can efficiently parasitize second‐instar *D. melanogaster* larvae, Ld exhibits superior host‐searching efficiency, enabling it to complete parasitism more rapidly.

We next conducted a competition experiment in which second‐instar *D. melanogaster* host larvae were exposed to both Aj and Ld at a 1:1:10 parasitoid‐parasitoid‐to‐host ratio for intervals of 20, 40, and 80 min (Figure 1C i). We found that the wasp emergence rates of Aj were significantly lower than those of Ld at all time points (Figure 1C ii). These results suggest that Aj presents a competitive disadvantage against Ld when encountering second‐instar young hosts, primarily due to the ability of Ld to locate hosts more rapidly. This finding raises the key question of how the weaker competitor, Aj, manages to coexist with Ld in the same habitat.

### 2.2 Aj Mitigates Competition with Ld by Expanding its Niche to Older Hosts

Host range expansion has been recognized as a fundamental adaptive strategy used by parasitoid wasps to mitigate interspecific competition.^[^ ^32^, ^36^, ^37^ ^]^ On the basis of this perspective, we hypothesized that Aj may have evolutionarily extended its host range to minimize direct competition with Ld. To test this hypothesis, we assessed the parasitism efficacy of Aj and Ld across nine *Drosophila* host species, including six from the *melanogaster* subgroup (*D. melanogaster*, *D. sechellia*, *D. santomea*, *D. simulans*, *D. yakuba*, and *D. erecta*) and three species outside this subgroup (*D. suzukii*, *D. pseudoobscura*, and *D. virilis*). We found that both Aj and Ld exhibited high wasp emergence rates, ranging from 64% to 91% for Ld and from 45% to 74% for Aj. Notably, Aj showed slightly lower parasitism efficacy than Ld in five host species (*D. melanogaster*, *D. simulans*, *D. yakuba*, *D. suzukii*, and *D. pseudoobscura*) (Figure S2, Supporting Information). These results indicate that Aj and Ld are generalist parasitoids with overlapping host ranges, suggesting that Aj may adopt alternative strategies, rather than host range expansion, to avoid competition with Ld.

To further investigate the mechanisms enabling Aj to coexist with Ld, we conducted parasitism efficacy experiments following a protocol similar to that described previously but using third‐instar *D. melanogaster* larvae as hosts (older hosts) (Figure 1A). These experiments included both non‐competition (Aj or Ld alone) (Figure 1B iii) and competition (Aj and Ld together) settings (Figure 1C iii). We observed that Ld often triggered strong defensive rolling behaviour in older hosts, which significantly impeded Ld's ability to oviposit (Movie S3, Supporting Information). Consistent with this, Ld exhibited extremely low wasp emergence rates in non‐competition experiments, with values of only 0.4%, 1% and 4% at 5, 10, and 20‐min intervals, respectively, and less than 11% even after 120 min (Figure 1B iii). In contrast, Aj successfully oviposited in older hosts (Movie S4, Supporting Information), achieving high wasp emergence rates of 21% at the 20‐min interval and exceeding 55% after 80 min—which is comparable to its performance with second‐instar hosts (younger hosts) (Figure 1B iii). Competition experiments further confirmed the superior parasitism efficiency of Aj, demonstrating its competitive advantage over Ld in third‐instar hosts at all the examined time points (Figure 1C iii). These results indicate that Aj achieves coexistence with Ld by partitioning host resources across different developmental stages, specifically targeting older hosts that are less susceptible to Ld parasitization.

### 2.3 Aj Parasitization Causes Host Imaginal Disc Degradation via Apoptosis

**Figure 2:**
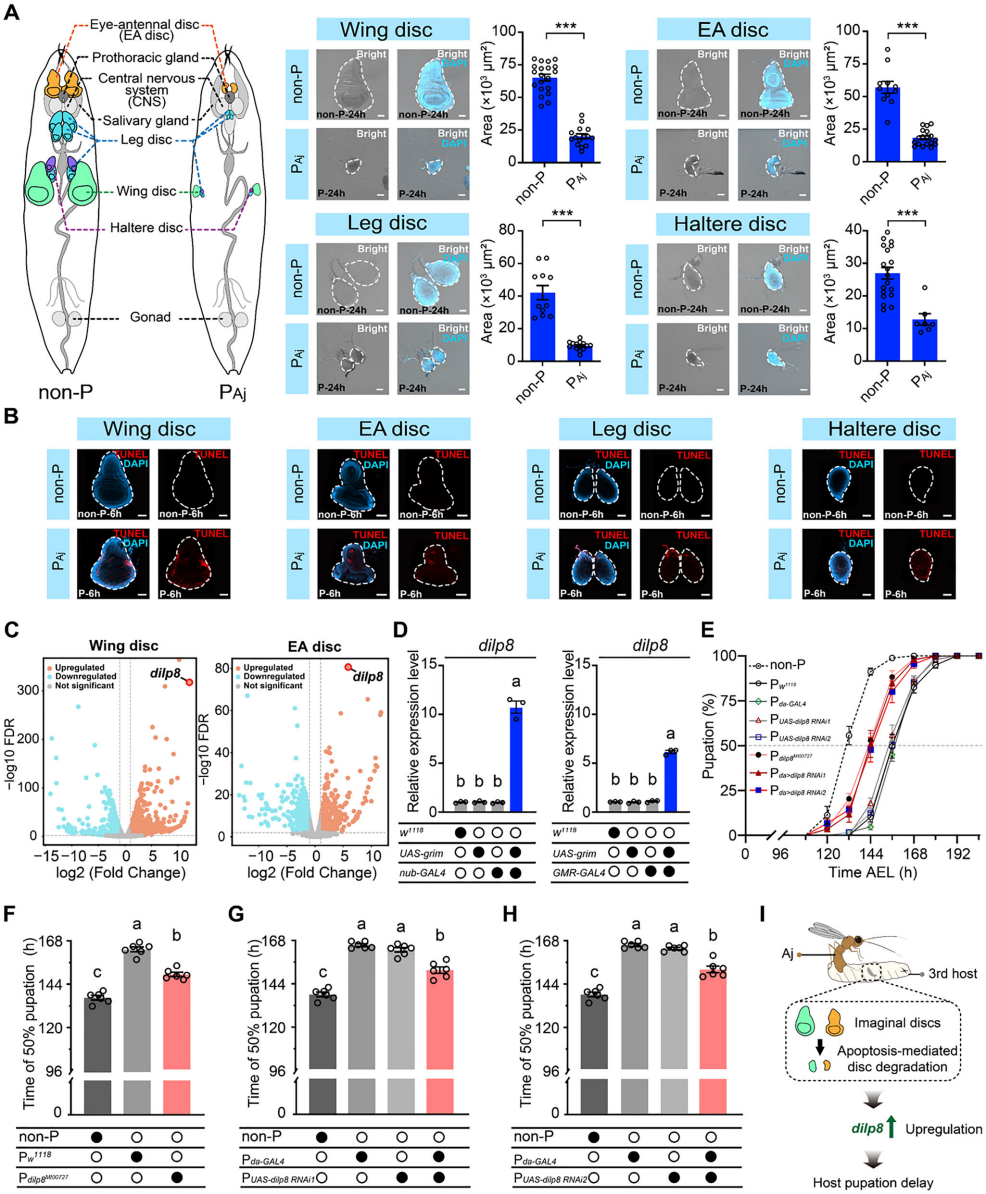
Aj parasitization induces apoptosis‐mediated disc degradation and causes host pupation delay. A) The sizes of imaginal discs, including wing discs, eye‐antennal (EA) discs, leg discs, and haltere discs, in non‐parasitized (non‐P) and Aj‐parasitized (P_Aj_) 3rd‐instar host larvae at 24 h post‐parasitization (P‐24h). The bright field image is shown in the Bright channel, while the nuclei of the host imaginal discs are labelled with DAPI (blue). The dashed lines mark the outlines of the imaginal discs. At least 7 imaginal discs of each type were analysed for size. The data are presented as the mean ± SEM. Statistical analysis was performed using a two‐tailed unpaired Student's *t* test when parametric assumptions and homogeneity of variances were met; Welch's *t* test was used to determine significance when parametric assumptions were met but heterogeneity of variances was observed. The Mann‒Whitney U test was used to determine significance when experiments required a nonparametric statistical test (^***^, *p <* 0.001). Scale bars: 50 µm. B) Cell apoptosis was detected via TUNEL (red) in imaginal discs of non‐P and P_Aj_ 3rd‐instar host larvae at 6 h post‐parasitization (P‐6h). The nuclei of the host imaginal discs are labelled with DAPI (blue). At least 30 imaginal discs of each type were examined. The dashed lines mark the outlines of the discs. Scale bars: 50 µm. C) Volcano plot of differentially expressed genes (DEGs) in the wing disc and EA disc between non‐P and P_Aj_ hosts. Each point in the volcano diagram represents one gene, and only those with |log_2_ (fold change)| > 1 and FDR < 0.01 were identified as DEGs. The red dots represent the upregulated DEGs, the blue dots represent the downregulated DEGs, and the gray dots represent the genes that were not significant. D) Relative expression levels of *dilp8* when *grim* was overexpressed in wing discs and EA discs. Three biological replicates were performed. Data are presented as the mean ± SEM. Significance was determined by one‐way ANOVA with Sidak's multiple comparisons test. Different letters indicate statistically significant differences (*p* < 0.05). E) Pupation timing curves of non‐P and Aj‐parasitized host larvae with different genotypes. The dashed line indicates the 50% pupation rate. Six biological replicates were performed. F) Time of 50% pupation of the non‐P and Aj‐parasitized *w^1118^* (P*_w_^1118^*) and *dilp8^MI00727^* (P*_dilp8_^MI00727^*) hosts tested in (E). G) Time of 50% pupation of non‐P and Aj‐parasitized *da‐GAL4* (P*_da‐GAL4_*), *UAS‐dilp8 RNAi1* (P*_UAS‐dilp8 RNAi1_*) and *da>dilp8 RNAi1* (P*_da>dilp8 RNAi1_*) hosts tested in (E). H) Time of 50% pupation of non‐P and parasitized *da‐GAL4* (P*_da‐GAL4_*), *UAS‐dilp8 RNAi2* (P*_UAS‐dilp8 RNAi2_*) and *da>dilp8 RNAi2* (P*_da>dilp8 RNAi2_*) hosts tested in (E). Data are presented as the mean ± SEM. The same values of the non‐P and P*_da‐GAL4_* groups were used in (F–H). Significance was determined by one‐way ANOVA with Sidak's multiple comparisons test. Different letters indicate statistically significant differences (*p* < 0.05). In (D) and (F–H), filled black circles represent the presence of a given transgene, and empty black circles represent the absence of a given transgene. I) Schematic diagram showing that Aj parasitization triggers apoptosis‐mediated degradation in host imaginal discs, leading to elevated levels of *dilp8*, a key regulator of developmental timing, which ultimately delays host pupation.

To understand how Aj successfully parasitizes older hosts, we performed parasitization experiments with third‐instar *D. melanogaster* larvae and dissected the hosts at 24 h post‐parasitization. Compared with those of non‐parasitized hosts, all imaginal discs (adult precursor tissues), including wing, eye‐antennal (EA), leg, and haltere discs, were drastically reduced in size (**Figure** **2**A). In contrast, other host tissues, such as the central nervous system (CNS), salivary gland, prothoracic gland, and gonads, remained unaffected and were comparable between parasitized and non‐parasitized host larvae (Figure S3, Supporting Information). These results demonstrate that Aj selectively targets and reduces the size of host imaginal discs while leaving non‐imaginal disc tissues intact.

We next used *nub‐GAL4>UAS‐EGFP* to specifically label wing discs with enhanced green fluorescent protein (EGFP) and monitored their morphology at distinct time points post‐parasitization (0, 6, and 24 h) in vivo. We found that Aj parasitization caused a significant reduction in the size of host wing imaginal discs by 24 h, whereas no detectable morphological changes were detected at 0 or 6 h compared with non‐parasitized controls (Figure S4A, Supporting Information). In addition, the wing discs of non‐parasitized hosts exhibited continuous growth over the time course (Figure S4B, Supporting Information). In contrast, the size of wing discs in Aj‐parasitized hosts at 24 h was significantly smaller than that at both 0 and 6 h (Figure S4B, Supporting Information). These results suggest that the observed reduction in imaginal disc size in parasitized older hosts is likely due to tissue degradation rather than developmental arrest. As expected, the terminal deoxynucleotidyl transferase‐mediated dUTP nick end labelling (TUNEL) assay revealed severe apoptotic signals in the imaginal discs, including the wing, EA, leg, and haltere discs, as early as 6 h post‐parasitization (Figure 2B). These findings reveal that Aj parasitization leads to the degradation of imaginal discs in parasitized hosts by inducing cell apoptosis.

### 2.4 Aj Parasitization Delays Host Development via Apoptosis‐Induced *dilp8* Upregulation

We collected wing and EA discs from both non‐parasitized hosts and Aj‐parasitized third‐instar *D. melanogaster* hosts at 6 h post‐parasitization for transcriptome analysis (Figure 2C). Comparative RNA profiling revealed 1639 and 844 genes with significantly greater expression in wing and EA discs of Aj‐parasitized hosts than in those of non‐parasitized hosts, respectively (Tables S1 and S2, Supporting Information). Among these genes, the *dilp8* gene has attracted particular attention because of its remarkable upregulation in Aj‐parasitized hosts, ranking second among upregulated genes in wing discs and first in EA discs compared with non‐parasitized hosts (Figure 2C). Subsequent qRT‒PCR experiments further confirmed these findings, revealing significantly elevated *dilp8* expression levels in the wing and EA discs of Aj‐parasitized hosts relative to those of non‐parasitized hosts (Figure S5, Supporting Information).

To determine whether the upregulation of *dilp8* is caused by imaginal disc apoptosis, we ectopically expressed *grim* (*UAS‐grim*), an activator of apoptosis, under the control of either wing‐specific (*nub‐GAL4*) or eye‐specific (*GMR‐GAL4*) drivers to induce apoptosis in these imaginal discs (Figure S6, Supporting Information). We observed a significant increase in *dilp8* expression in both *nub>grim* and *GMR>grim* larvae (Figure 2D). Interestingly, we also found that Aj parasitization caused an obvious delay in pupation among parasitized host larvae, with an average delay of 26.36 h compared with that of non‐parasitized hosts (Figure 2E,F). To confirm that the upregulation of *dilp8* is responsible for this developmental delay, we performed two independent experiments in which hosts with decreased *dilp8* levels were used. First, we used a hypomorphic mutation (*dilp8^MI00727^*), which significantly reduced the pupation delay induced by Aj‐parasitization compared with that in *w^1118^* controls (Figure 2E,F). Second, we used two RNAi lines (*UAS‐dilp8 RNAi1* and *UAS‐dilp8 RNAi2*) to generate *dilp8*‐knockdown hosts using a ubiquitous GAL4 driver (*da‐GAL4*). Compared with the parasitized controls (*da‐GAL4* alone, *UAS‐dilp8 RNAi1* alone, and *UAS‐dilp8 RNAi2* alone), both the *da>dilp8 RNAi1* and *da>dilp8 RNAi2* hosts exhibited significantly shorter pupation delays after parasitization (Figure 2E,G,H). These results indicate that Aj parasitization induces pupation delay in third‐instar *D. melanogaster* hosts, and this process is mediated by increased levels of *dilp8* resulting from imaginal disc apoptosis (Figure 2I).

### 2.5 Venom is Essential for Triggering the Apoptosis‐Mediated Degradation of Host Imaginal Discs

**Figure 3:**
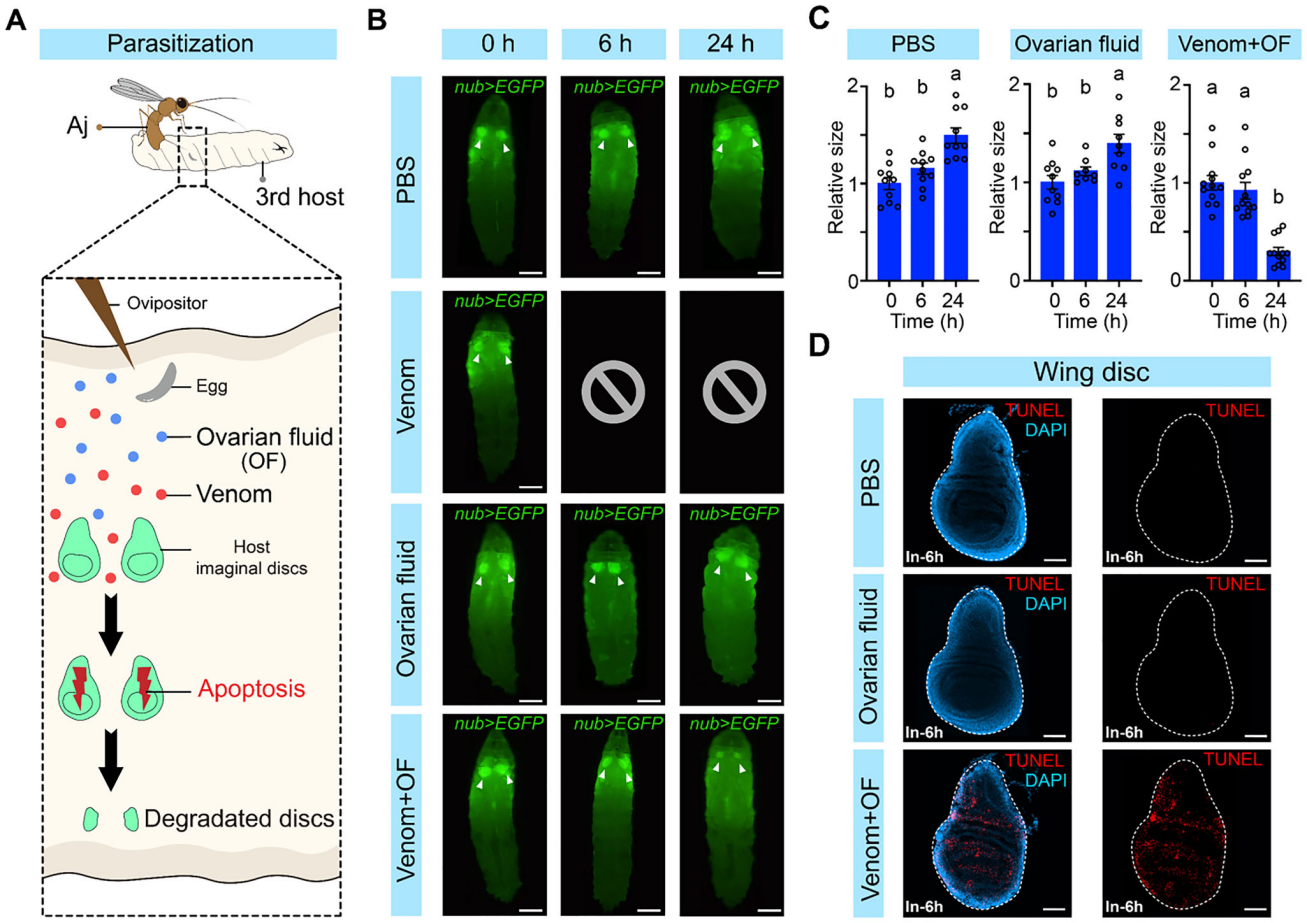
Venom is required to induce apoptosis‐mediated degradation of host imaginal discs. A) Schematic diagram of different parasitic factors released into host larvae during Aj parasitization and their possible roles in inducing apoptosis‐mediated degradation of host imaginal discs. B) Host wing discs (indicated by white arrowheads) labelled with *nub>EGFP* (green) were examined at 0, 6, and 24 h following different injection treatments: 1 × PBS injection (PBS), venom injection (Venom), ovarian fluid injection (Ovarian fluid), and venom plus ovarian fluid injection (Venom+OF). At least 50 *Drosophila* 3rd‐instar larvae from each treatment group were examined at each time point. Scale bars: 500 µm. C) The relative size of host wing discs at 0, 6, and 24 h post‐injection with different treatments in (B) was measured. At least 8 wing discs of each type were analysed for size. Data are presented as the mean ± SEM. Significance analysis was performed using one‐way ANOVA with Sidak's multiple comparisons test. Different letters indicate statistically significant differences (*p* < 0.05). D) Wing discs of 3rd‐instar host larvae were dissected at 6 h post‐injection in (B). At least 30 imaginal discs from each treatment group were examined. The apoptotic cells were subjected to TUNEL (red), and the nuclei were labelled with DAPI (blue). The dashed lines mark the outlines of the wing discs. Scale bars: 50 µm.

PDVs and teratocytes have not been identified in *Asobara* species, including Aj. As such, Aj mainly relies on venom, ovarian fluid, and larval secretions as parasitic effectors to manipulate host physiology.^[^ ^40^, ^41^ ^]^ Venom and ovarian fluid are co‐injected into hosts during Aj oviposition, whereas larval secretions are produced by wasp larvae after egg hatching (**Figure** **3**A). Since Aj eggs require ≈40 h to hatch, only venom and/or ovarian fluid are present during the early stages of parasitization and potentially drive apoptosis‐induced degradation of host imaginal discs. We then microinjected third‐instar host larvae with venom, ovarian fluid, or a combination of venom and ovarian fluid to monitor the fate of their imaginal discs. Consistent with previous reports, venom injection alone resulted in 100% mortality (50/50) in *D. melanogaster* hosts, whereas ovarian fluid neutralized the strong toxicity of venom, allowing hosts to survive normally.^[^ ^41^, ^42^ ^]^ Interestingly, we found that injection of a combination of venom and ovarian fluid induced significant degradation of host wing imaginal discs at 24 h post‐injection (Figure 3B,C), with apoptosis detected in the discs as early as 6 h post‐injection, compared with the 1 × PBS‐injected controls (Figure 3D). This phenotype closely mirrored that observed in Aj‐parasitized third‐instar hosts (Figure 2B; Figure S4, Supporting Information). In contrast, the injection of ovarian fluid alone had no detectable effects on the host imaginal discs (Figure 3B–D). Collectively, these results indicate that Aj venom is responsible for the apoptosis‐mediated degradation of imaginal discs in parasitized *D. melanogaster* hosts.

### 2.6 Identification of the Aj Venom Protein Repertoire

We then asked which venom component plays the crucial role in inducing host imaginal disc degradation. To establish a comprehensive catalogue of Aj venom proteins, we first sequenced and *de novo* assembled the Aj reference genome based on 32.4 Gb of PacBio long‐read sequencing data and 24.08 Gb of Illumina paired‐end sequencing data (Table S3, Supporting Information). The assembled genome was 273.7 Mb, exhibiting high contiguity with an N50 size of 16.8 Mb (Table S4, Supporting Information). BUSCO analysis revealed a high level of completeness (99.1%) (Table S4, Supporting Information). By integrating 65.22 Gb of Hi‐C data, 98.3% of the assembled contigs were anchored to seventeen pseudochromosomes, which was consistent with the karyotype staining results (Tables S3 and S4 and Figure S7, Supporting Information). Using a combination of ab initio gene predictions, transcriptomic evidence, and homologous alignments, we generated an official gene set consisting of 13931 protein‐coding genes for Aj (Table S4, Supporting Information). We then sequenced the transcriptome of Aj venom glands (VGs) and characterized 2333 genes within the gene set with robust expression evidence in VG (Figure S8, Supporting Information). Given that not all expressed genes are translated into venom proteins (VPs) in VG and stored in the venom reservoir (VR), we extracted venom from Aj VRs and performed LC‒MS/MS to further characterize reliable VPs (Figure S8A, Supporting Information). In total, we identified 186 genes encoding reliable VPs, supported by both transcriptomic and proteomic evidence, and all of which possess signal peptides (Figure S8B and Table S5, Supporting Information).

### 2.7 Knockdown of VID‐1 Prevents the Apoptosis‐Mediated Degradation of Host Imaginal Discs

**Figure 4:**
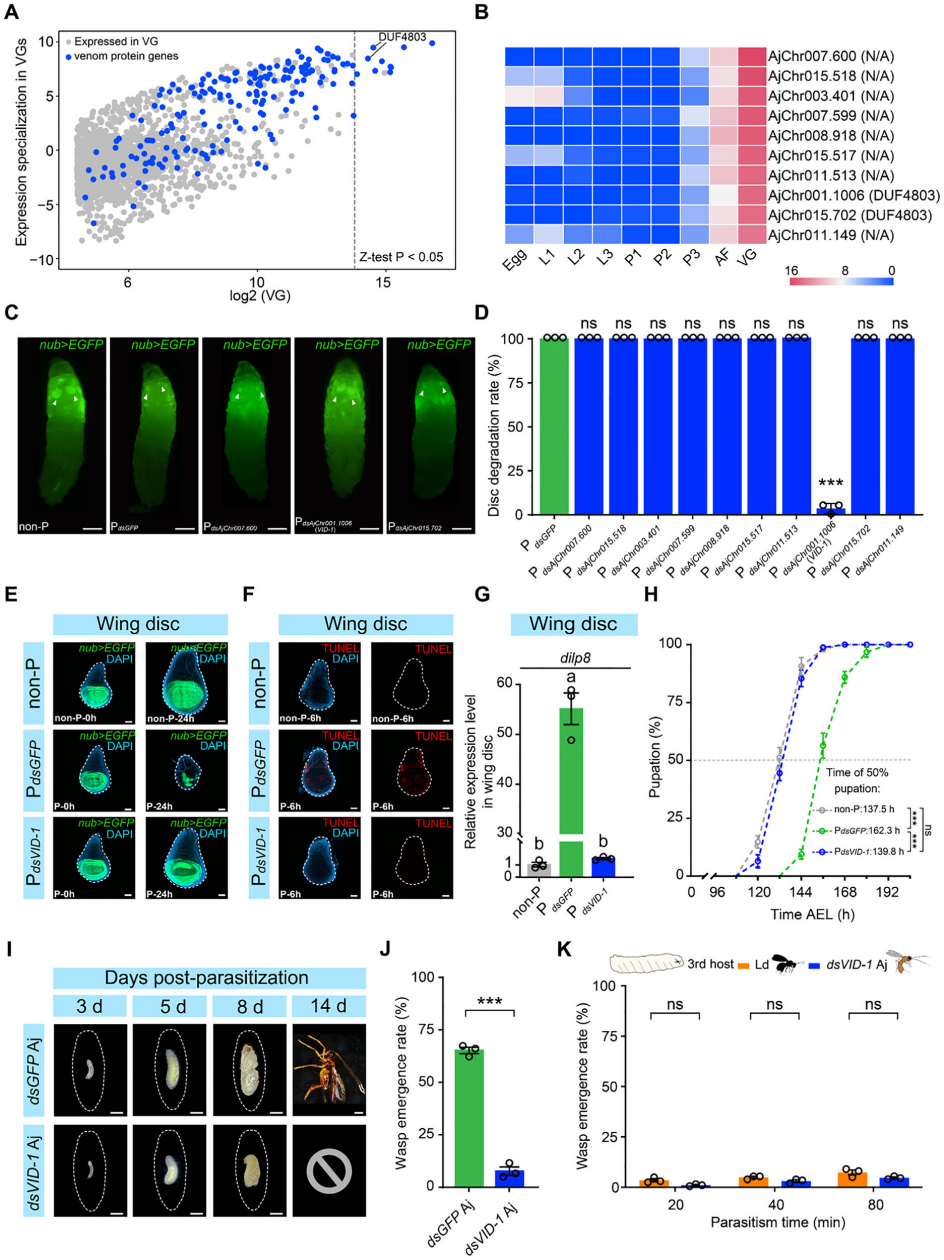
The *VID‐1* venom protein gene facilitates Aj parasitism in older hosts. A) Identification of Aj venom protein (VP) genes. The expression values in Aj venom glands (VGs) and the specialized levels of expression in VGs are presented on the *x*‐axis and *y*‐axis, respectively. All the genes expressed in VGs (expression level ≥ 6.91) are shown in the figure. The blue dots represent the VP genes. The full VP gene list is provided in Table S5 (Supporting Information). B) Transcription heatmap of the top 10 highly expressed VP genes. The genes are displayed in order of their expression levels in the VG. C) Host wing discs (indicated by white arrowheads) labelled with *nub>EGFP* (green) were examined for apoptosis‐mediated degradation in both non‐P and Aj‐parasitized 3rd‐instar larvae at 24 h post‐parasitization. The parasitized groups included 4 *dsRNA*‐treated conditions: P*_dsGFP_*, P*_dsAjChr007.600_*, P*_dsAjChr001.1006_*, and P*_dsAjChr015.702_*. Sixty *Drosophila* larvae from each treatment group were examined. Scale bars: 500 µm. D) Disc degradation rate of *Drosophila* 3rd‐instar host larvae that were parasitized by *dsRNA‐*treated Aj. P*_dsGFP_* was used as a control. Three biological replicates were performed. Data are presented as the mean ± SEM. Significance was determined by Welch's *t* test (ns, not significant; ^***^, *p* < 0.001). E) Wing discs of non‐P and parasitized 3rd‐instar host larvae at 0 and 24 h post‐parasitization by *dsGFP‐*treated Aj (P*_dsGFP_*) or *dsVID‐1*‐treated Aj (P*_dsVID‐1_*). The non‐P and P*_dsGFP_* served as controls. At least 30 imaginal discs from each treatment group were examined. The nuclei were stained with DAPI (blue), and the wing discs were labelled with *nub>EGFP* (green). The dashed lines mark the outlines of the wing discs. Scale bars: 50 µm. F) Wing discs of non‐P and parasitized 3rd‐instar host larvae were dissected at 6 h post‐parasitization by *dsGFP‐*treated Aj (P*_dsGFP_*) or *dsVID‐1*‐treated Aj (P*_dsVID‐1_*). At least 30 imaginal discs from each treatment group were examined. Cell apoptosis was detected via TUNEL (red), and nuclei were stained with DAPI (blue). The dashed lines mark the outlines of the wing discs. Scale bars: 50 µm. G) Relative expression level of *dilp8* in the wing discs of P*_dsVID‐1_* 3rd‐instar host larvae at 6 h post‐parasitization compared with P*_dsGFP_* and non‐P controls. Three biological replicates were performed. Data are presented as the mean ± SEM. Significance was determined by one‐way ANOVA with Sidak's multiple comparisons test. Different letters indicate statistically significant differences (*p* < 0.05). H) Pupation timing curves of non‐P (gray curve), P*_dsGFP_* (green curve), and P*_dsVID‐1_* (blue curve) 3rd‐instar host larvae. Six biological replicates were performed. I) Representative images of different developmental stages of *dsGFP‐*treated Aj and *dsVID‐1‐*treated Aj at 3, 5, 8, and 14 d post parasitization. The dashed lines mark the outline of the host. Scale bars: 500 µm. J) Wasp emergence rate of *dsGFP*‐treated Aj and *dsVID‐1*‐treated Aj. Three biological replicates were performed. Data are presented as the mean ± SEM. Significance was determined by a two‐tailed unpaired Student's *t* test (^***^, *p <* 0.001). K) Wasp emergence rate of *dsVID‐1‐*treated Aj and Ld when parasitizing 3rd‐instar hosts in competition experiments at an Aj:Ld:host ratio of 1:1:10 at 20, 40, and 80 min. Data are presented as the mean ± SEM. Three biological replicates were performed for each time point. Significance was analysed by two‐way ANOVA with Sidak's multiple comparisons test (ns, not significant).

Like the VP genes of other parasitoid wasps, a large proportion of the 186 identified Aj VP genes encode proteins with uncharacterized functions (**Figure** **4**A; Table S5, Supporting Information). We thus prioritized the top 10 most highly expressed VP genes for further investigation, including 2 genes (*AjChr001.1006* and *AjChr015.702*) each containing a domain of unknown function (DUF4803) and 8 genes lacking any domains (Figure 4A,B). Notably, all 10 genes presented remarkable expression specialization in the VG (Figure 4B). We next performed in vivo RNA interference (RNAi) experiments to explore the roles of these highly expressed VP genes in the degradation of host imaginal discs (Figure S9A, Supporting Information). Quantitative real‐time PCR (qRT‒PCR) confirmed that the expression levels of these genes were significantly reduced in Aj adults after the injection of the corresponding double‐stranded RNA (dsRNA) into Aj prepupae (Figure S9B, Supporting Information). The resulting dsRNA‐treated Aj females were then used to parasitize *nub‐GAL4>UAS‐EGFP* third‐instar hosts and assess wing imaginal disc degradation. Notably, knockdown of *AjChr001.1006* resulted in a significantly lower host disc degradation rate of only 3.3% (2/60) (Figure 4C,D). In contrast, parasitization by *dsGFP*‐injected control wasps and wasps with knockdown of the other 9 tested genes, including *AjChr015.702* (which also contains DUF4803), caused 100% host disc degradation (60/60) (Figure 4C,D; Figure S10, Supporting Information). We further dissected wing imaginal discs from *nub‐GAL4>UAS‐EGFP* hosts and assessed their apoptotic signals using TUNEL assays. We found that, compared with non‐parasitized host larvae, parasitization by *dsGFP*‐injected wasps induced apoptosis at 6 h post‐parasitization and caused significant degradation of host wing discs at 24 h post‐parasitization (Figure 4E,F). In contrast, parasitization by *dsAjChr001.1006*‐injected wasps failed to induce apoptosis at 6 h post‐parasitization and caused no detectable morphological changes in the host wing discs at 24 h post‐parasitization (Figure 4E,F). Based on its identity as a VP gene and its ability to induce tissue degradation, we designated *AjChr001.1006* as the Venom‐Induced Degradation in host imaginal disc (*VID‐1*) gene. Collectively, these results indicate that *VID‐1* is responsible for the apoptosis‐mediated degradation of host imaginal discs.

### 2.8 VID‐1 Induces Host Developmental Delay and Facilitates Aj Parasitism in Older Hosts

Our previous findings demonstrated that Aj parasitization causes host imaginal disc degradation via apoptosis, which in turn induces *dilp8* upregulation and further delays host pupation (Figure 2). Given that the venom protein *VID‐1* is responsible for the apoptosis‐mediated degradation of host imaginal discs, we asked whether the knockdown of *VID‐1* would also suppress *dilp8* induction and rescue the host developmental delay phenotype. To address this question, we utilized either *dsGFP*‐injected control wasps or *dsVID‐1*‐injected wasps to parasitize third‐instar host larvae. Compared with non‐parasitized hosts, *dsGFP*‐injected wasp parasitization resulted in significantly elevated *dilp8* expression in host imaginal discs and delayed pupation (Figure 4G,H; Figure S11, Supporting Information). Strikingly, *dsVID‐1* knockdown completely suppressed *dilp8* induction in parasitized host imaginal discs (Figure 4G; Figure S11, Supporting Information). Furthermore, the parasitization‐induced developmental delay was fully rescued in hosts parasitized by *dsVID‐1*‐injected wasps, with the timing of pupation being indistinguishable from that of non‐parasitized hosts (Figure 4H).

Pupation delay of parasitized hosts may provide crucial developmental time for wasp offspring by extending the host larval period. We therefore investigated whether abolishing this delay through *VID‐1* knockdown would impair wasp development. We observed that *VID‐1* knockdown severely impaired Aj offspring development. Specifically, wasp offspring in hosts parasitized by *dsVID‐1*‐injected wasps were significantly smaller than those in hosts parasitized by *dsGFP*‐injected controls at 3–5 days post‐parasitization (Figure 4I). While offspring of *dsGFP*‐injected wasps pupated by Day 8 and emerged as adults by Day 14, most offspring of *dsVID‐1*‐injected wasps died by Day 8 without successful eclosion (Figure 4I). Consistent with these developmental defects, we observed significantly lower wasp emergence rates for *dsVID‐1*‐injected wasps than for *dsGFP*‐injected controls (Figure 4J). Competition experiments further revealed that Aj lost its competitive advantage over Ld in third‐instar hosts when the VP gene *VID‐1* was knocked down (Figure 4K). Collectively, these results indicate that *VID‐1* induces host developmental delay through apoptosis‐mediated *dilp8* upregulation, enabling the successful development of Aj offspring in older hosts and enhancing its competitive advantage.

### 2.9 The Functionalization of Expanded DUF4803‐Domain Genes Yields Five Venom VIDs that Degrade Host Imaginal Discs

**Figure 5:**
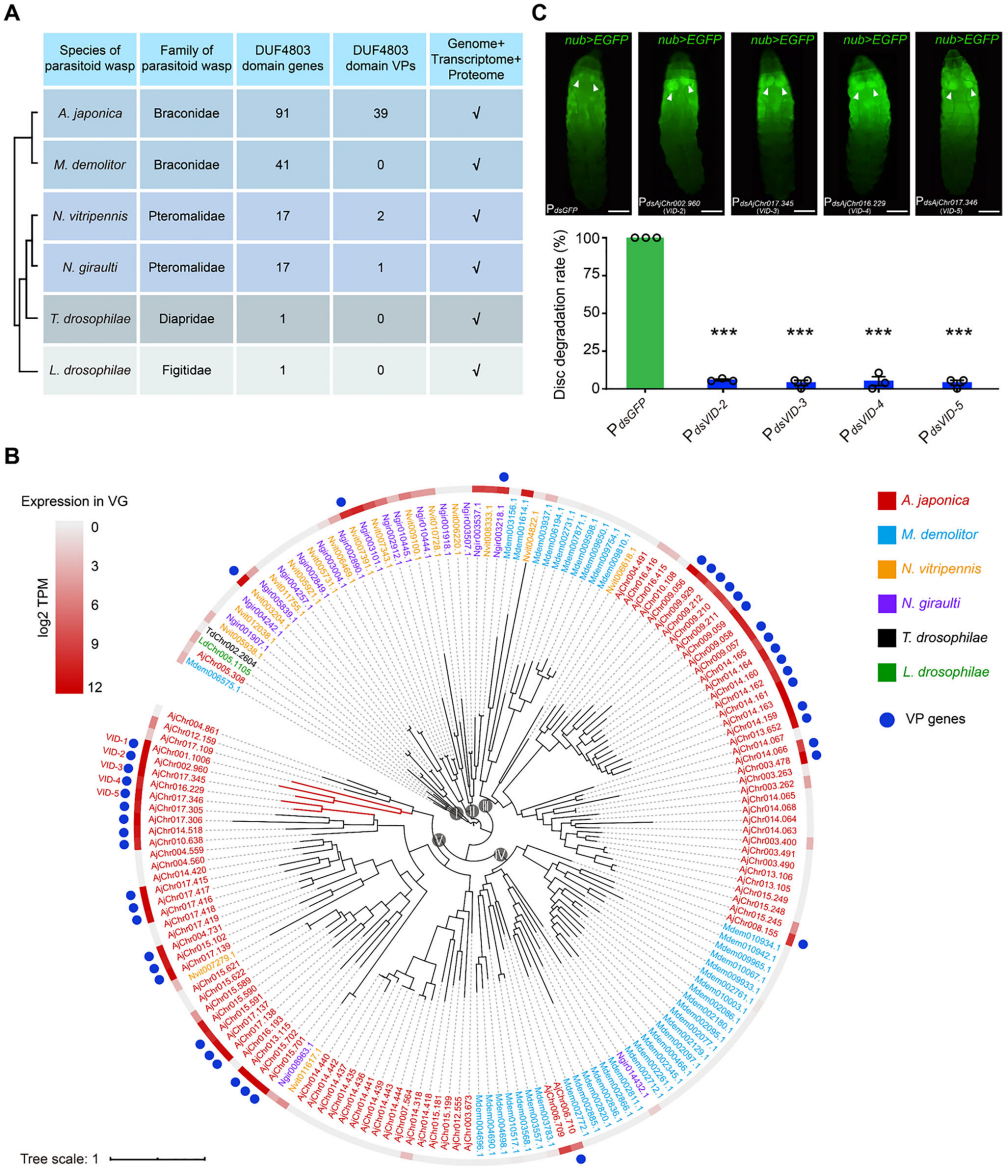
Evolution of DUF4803‐domain genes in parasitoid wasps. A) Expansion information of DUF4803‐domain genes in 6 parasitoid species with high‐quality genomes, venom gland transcriptomes, and venom proteomic data. The numbers of DUF4803‐domain genes and DUF4803‐domain VP genes are shown in the table. The expansion information of DUF4803‐domain genes in the other 10 parasitoid species with high‐quality genomes but lacking venom gland transcriptomes or proteomic data is shown in Figure S12 (Supporting Information). B) Maximum‐likelihood phylogenetic tree of DUF4803‐domain genes among the 6 parasitoid species. Different species are represented in different colours. The heatmap outside the tree indicates the expression levels of DUF4803‐domain genes in the venom gland. The blue points indicate the identified VP genes. C) Disc degradation rate of 3rd‐instar host larvae that were parasitized by *dsRNA‐*treated Aj. The parasitized groups included different *dsRNA*‐treated conditions: P*_dsGFP_*, P*_dsAjChr002.960_*_(_ *_VID‐2_*_)_, P*_dsAjChr017.345_*_(_ *_VID‐3_*_)_, P*_dsAjChr016.229_*_(_ *_VID‐4_*_)_, and P*_dsAjChr017.346_*_(_ *_VID‐5_*_)_. P*_dsGFP_* was used as a control. Data are presented as the mean ± SEM. Three biological replicates were performed. Significance was determined by one‐way ANOVA with Sidak's multiple comparisons test (^***^, *p* < 0.001). Representative images of 3rd‐instar *nub>EGFP Drosophila* host larvae at 24 h post‐parasitization are shown. Scale bars: 500 µm. *A. japonica*, *Asobara japonica*; *M. demolitor*, *Microplitis demolitor*; *N. vitripennis*; *Nasonia vitripennis*; *N. giraulti*, *Nasonia giraulti*; *T. drosophilae*, *Trichopria drosophilae*; *L. drosophilae*, *Leptopilina drosophilae*.

Given the functional significance of *VID‐1*, a DUF4803‐domain VP gene, we analysed the evolution of DUF4803‐domain genes across parasitoid wasps using species with high‐quality genomes, venom gland transcriptomes, and venom proteomic data to decipher the adaptive history of this gene family in venom systems. We systematically screened for DUF4803‐domain genes across six parasitoid wasp species, including Aj and *Microplitis demolitor* (Md) from Braconidae, *Nasonia vitripennis* (Nv) and *N. giraulti* (Ng) from Pteromalidae, *Trichopria drosophilae* (Td) from Diapriidae, and Ld from Figitidae (**Figure** **5**A). We found a remarkable expansion of DUF4803‐domain genes in Braconidae, with significantly greater copy numbers (Aj: 91; Md: 41) than those in other families (Nv: 17; Ng: 17; Td: 1; Ld: 1) (Figure 5A). This expansion pattern was consistently observed in our extended analysis of additional parasitoid wasp species that had high‐quality genomes but lacked comprehensive venom gland transcriptomes or proteomic data (Figure S12, Supporting Information). Phylogenetic analysis of DUF4803‐domain genes across the six parasitoid wasp species resulted in five main clades (Figure 5B). The basal clade (clade I) contained orthologous genes present in all analysed species, reflecting their ancestral status prior to lineage diversification (Figure 5B). The other clades exhibited lineage‐specific expansions, i.e., clade II underwent expansion exclusively in Nasonia species, clade III expanded in both Aj and Md, clade IV showed Md‐specific duplications, and clade V displayed Aj‐specific expansions (Figure 5B). Importantly, a substantial proportion of Aj's DUF4803‐domain genes in clades III and V exhibited high expression in venom glands and were recruited as VP genes (Figure 5B). Notably, Aj has recruited 39 VP genes among its 91 DUF4803‐domain genes (17 in clade III, 1 in clade IV, and 21 in clade V), whereas its competitor Ld retains only a single non‐venom DUF4803‐domain gene (Figure 5B; Figure S13, Supporting Information).

Our functional analysis revealed that although both *VID‐1* (*AjChr001.1006*) and *AjChr015.702* encode DUF4803‐domain proteins within clade V, only *VID‐1* exhibited the capacity to degrade host imaginal discs (Figure 4C,D). This functional divergence prompted a comprehensive RNAi screen targeting the remaining 19 DUF4803‐domain VP genes in clade V to identify additional disc degradation effectors (Figure 5B). The qRT‒PCR results revealed significant knockdown of these DUF4803‐domain genes in emerging Aj adults following dsRNA injection at the prepupal stage (Figure S14, Supporting Information). As in our previous experiments, dsRNA‐treated Aj females were subsequently used to parasitize *nub‐GAL4>UAS‐EGFP* third‐instar host larvae, with *dsGFP*‐injected female wasps serving as controls. While parasitization by *dsGFP*‐injected control wasps and wasps with the knockdown of 15 other DUF4803‐domain genes resulted in 100% host disc degradation, the knockdown of 4 specific genes (*AjChr002.960*, *AjChr017.345*, *AjChr016.229*, and *AjChr017.346*) significantly suppressed degradation rates (Figure 5C; Figure S15, Supporting Information). These genes were thus termed *VID‐2* (*AjChr002.960*), *VID‐3* (*AjChr017.345*), *VID‐4* (*AjChr016.229*), and *VID‐5* (*AjChr017.346*) according to their functional similarity to *VID‐1* in mediating host disc degradation. In addition, knockdown of any individual *VID* gene did not affect the expression levels of other *VID* genes, suggesting that all five *VID* genes are jointly required for host disc degradation (Figure S16, Supporting Information).

**Figure 6:**
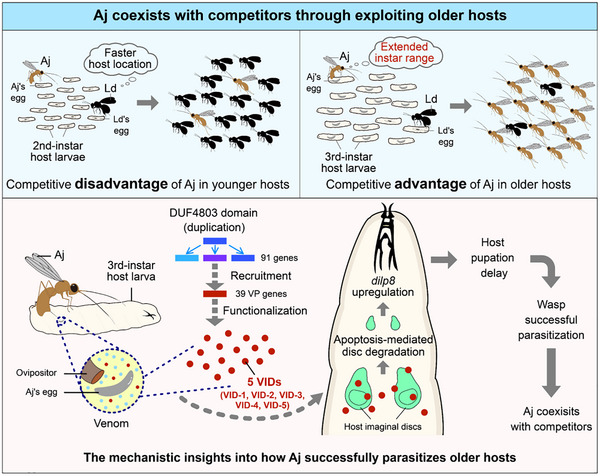
Proposed model of how Aj coexists with its competitors. Aj shows a competitive disadvantage against Ld in younger hosts (2nd‐instar larvae) because of the faster host location efficiency of Ld. In contrast, Aj has evolved to exploit older hosts (3rd‐instar larvae), allowing it to coexist with Ld via host resource partitioning. Five VID venom proteins (VID‐1–VID‐5) are injected into 3rd‐instar host larvae along Aj parasitization. The genes encoding this set of proteins ancestrally originated from the duplication event of DUF4803‐domain paralogs, with the Aj genome containing 91 DUF4803‐domain copies. Among them, 39 were recruited as VP genes, with 5 (*VID‐1*–*VID‐5*) ultimately being functionalized to induce the apoptosis‐mediated degradation of host imaginal discs. This process elevates *dilp8* expression, causing host developmental delay that enables Aj to successfully parasitize older hosts.

Collectively, these results indicate that DUF4803‐domain genes originated from their ancestral precursors and subsequently underwent multiple expansion events, predominantly within the Braconidae family. Unlike their orthologs in other parasitoid wasps, some DUF4803‐domain genes in Aj were recruited into the venom system as VP genes. Notably, five of these genes (*VID‐1*–*VID‐5*) evolved the unique capacity to induce apoptotic degradation of host imaginal discs—a derived trait that secures parasitic success by enabling the effective exploitation of older hosts, thereby facilitating competitive coexistence via the partitioning of host resources (**Figure** **6**).

## 3 Discussion

Interspecific competition is a fundamental driver that shapes ecological communities, particularly among species that occupy overlapping niches.^[^ ^1^, ^8^, ^9^, ^11^ ^]^ The prevailing ecological paradigm suggests that intense interspecific competition drives evolutionary adaptations that minimize direct resource competition, thereby promoting species coexistence.^[^ ^1^, ^15^, ^20^, ^23^ ^]^ Here, we used two competing parasitoids (Aj and Ld) as a model system to investigate coexistence strategies and the underlying mechanisms. Our results reveal that despite being generalist parasitoids with nearly identical host ranges (Figure S2, Supporting Information), they achieve coexistence through the partitioning of host resources: Aj has evolved to exploit older (third‐instar) hosts, whereas Ld maintains superiority in exploiting younger (second‐instar) hosts (Figure 1B,C). Host shift strategies, including switching to different host species or expanding host ranges, are considered the most prevalent adaptive solutions for parasitoid coexistence.^[^ ^32^, ^36^, ^37^ ^]^ Our study reveals an alternative strategy: host developmental‐stage partitioning. Unlike host shifting, this temporal niche partitioning minimizes evolutionary trade‐offs by leveraging pre‐existing host recognition systems while enabling rapid niche differentiation through temporal segregation rather than host taxonomic separation.

Our study provides mechanistic insights into how Aj successfully parasitizes older hosts to avoid interspecific competition and coexist with competitors. Both Aj and Ld are koinobiont endoparasitoids that lay eggs inside *Drosophila* host larvae. Their offspring complete development within the host body and emerge as adults during the host pupal stage.^[^ ^38^, ^39^ ^]^ Parasitizing older host larvae typically constrains wasp offspring development time within host larvae, resulting in mortality of wasp progeny. To overcome this limitation, Aj has evolved an innovative solution through venom‐mediated host developmental manipulation. That is, Aj employs 5 DUF4803‐domain VP genes (*VID‐1*–*VID‐5*) that induce apoptosis‐mediated degradation of host imaginal discs (Figures 4, 5), leading to elevated *dilp8* expression and subsequent host pupation delay (Figure 4G,H; Figure S11, Supporting Information). This process extends the host larval developmental period, thereby ensuring complete wasp offspring development and successful emergence (Figure 4I,J). It is noteworthy that parasitization‐mediated developmental manipulation is observed in some other systems, with several molecular mechanisms elucidated. For instance, the bracovirus of *Cotesia plutellae* encodes a viral histone H4 (CpBV‐H4) that suppresses host insulin signaling (PxILP1) and chromatin remodeling factors, thereby epigenetically altering the development of its host, *Plutella xylostella*, to extend the larval stage.^[^ ^43^, ^44^ ^]^ In *C. vestalis*, miRNAs derived from teratocytes (Cve‐miR‐281‐3p) and bracovirus (Cve‐miR‐novel22‐5p‐1) cooperatively target the host ecdysone receptor, inducing a developmental delay in *P. xylostella* larvae.^[^ ^45^ ^]^ Similarly, the endoparasitoid *C. sonorensis* employs ichnovirus‐encoded Cys‐motif proteins (VHv1.1) to induce developmental delays in its host *Heliothis virescens* by inhibiting translation.^[^ ^46^ ^]^ Furthermore, venom proteins alone can achieve similar outcomes, as demonstrated by a metalloproteinase (EpMP3) from *Eulophus pennicornis* that disrupts host pupation timing.^[^ ^47^ ^]^ Whether analogous regulatory mechanisms exist in the drosophilid parasitoids presents an intriguing avenue for future research. Furthermore, comparative venom proteomics revealed striking compositional differences between Aj and Ld, with the complete absence of DUF4803‐domain proteins in the venom arsenal of Ld (Figure S17, Supporting Information). Consistent with this difference, even on the rare occasions when Ld successfully oviposits in older hosts, it does not induce imaginal disc degradation (Figure 1B iii; Figure S18, Supporting Information). Notably, the contrasting strategies of Aj and Ld underscore a casade of adaptations necessary for niche partitioning. The primary adaptation is behavioral: Aj efficiently recognizes and oviposits in older hosts, a niche Ld fails to exploit due to effective host defenses (Movies S3 and S4, Supporting Information). The secondary, reinforcing adaptation is physiological: Aj employs a unique venom arsenal, including DUF4803‐domain proteins absent in Ld (Figure S17, Supporting Information), which is crucial for overcoming the developmental constraints of parasitizing older hosts. Consequently, Ld is limited to dominating younger hosts through superior host‐searching efficiency (Figure S1, Supporting Information), while Aj's integration of both behavioral and physiological adaptations secures its niche in older hosts.

Venom serves as an essential parasitic effector in nearly all parasitoid wasps, playing a crucial role in facilitating successful host exploitation.^[^ ^32^, ^33^, ^48^, ^49^, ^50^ ^]^ Recently, rapidly evolving parasitoid VP genes have emerged as excellent systems for studying evolutionary models of novel genes. While single‐copy gene co‐option represents a well‐documented recruitment mechanism in some parasitoids (e.g., *Nasonia vitripennis*), accumulating evidence highlights the importance of gene duplication events in VP gene evolution.^[^ ^50^ ^]^ These include (i) the duplicated *Lar* VP genes in *L. heterotoma* that suppress host cellular immunity,^[^ ^32^ ^]^ (ii) the extensive expansion of *Warm* VP genes in *L. boulardi* that enables evasion of host immune defences,^[^ ^32^ ^]^ (iii) the duplicated *EsGAP* VP genes in *L. boulardi* and *L. heterotoma* that trigger host escape behaviour,^[^ ^33^ ^]^ and (iv) the duplicated *TdTimp* VP genes in *Trichopria drosophilae* that induce developmental arrest in host larvae.^[^ ^31^ ^]^ Our study reveals another case of VP gene recruitment through duplication events in the Aj parasitoid wasp. Comparative genomic analysis showed remarkable expansion of DUF4803‐domain genes in Braconidae, particularly in Aj, which harbours 91 copies—the maximum observed across all analysed parasitoid species (Figure 5A; Figure S12, Supporting Information). Among them, 39 DUF4803‐domain genes were successfully recruited as VP genes, accounting for 21% (39/186) of the total VP genes in Aj (Figure 5A,B). This massive gene duplication and subsequent recruitment are essential for Aj to obtain the 5 DUF4803‐domain *VID* genes that enable parasitism of older hosts, thereby facilitating coexistence with its competitors (Figure 4K). These results exemplify how interspecific competition drives adaptive gene family expansion and functional innovation for niche differentiation. Notably, unlike previously reported cases in which parasitoids co‐opted duplicated genes containing known functional domains (e.g., RhoGAP, mucin‐bd, or Timp domains),^[^ ^31^, ^32^, ^33^ ^]^ the DUF4803 domain represents a domain of unknown function, highlighting the ability of parasitoids to functionalize even uncharacterized domain genes. Importantly, how this set of *VID* genes selectively induces host imaginal disc degradation, as well as the functional roles of the remaining 34 DUF4803‐domain VP genes in modulating host processes, warrants further investigation in future studies. Furthermore, an intriguing possibility arises from our competition experiments: the venom of Aj may directly affect the development of Ld offspring. This is suggested by the observed reduction in the emergence rate of Ld in competition versus non‐competition experiments (Figure 1B ii,C ii). A critical question for future research is whether this detrimental effect is mediated by the newly identified *VID* genes or by other VP genes.

In conclusion, our study demonstrated that sympatric parasitoid wasps can achieve coexistence through developmental stage‐specific host resource partitioning. This strategy involves an initial behavioral specialization toward a distinct host stage, followed by a venom‐based physiological adaptation necessary for successful parasitism. We elucidate the molecular mechanisms and evolutionary processes underlying this physiological adaptation, including the characterization of specific novel genes associated with competitive interactions. These findings advance the understanding of species coexistence mechanisms and contribute to the growing body of knowledge regarding the competitive niche shift principle.

## 4 Experimental Section

### Insects

The parasitoid wasps *A. japonica* (Aj)^[^ ^38^ ^]^ and *L. drosophilae* (Ld)^[^ ^39^ ^]^ were originally collected from the same *Myrica rubra* orchard in Taizhou, China (28°50′N, 120°34′E), during our 2018 field surveys. Aj is a thelytokous parthenogenetic species, whereas Ld is a sexual haplodiploid parasitoid wasp. Both species were maintained in the laboratory with *D. melanogaster* (*w^1118^* strain) as a regular host under controlled environmental conditions (25 °C, 50% relative humidity, 16:8 h light: dark cycle). Newly eclosed adult wasps were provided with apple juice agar medium (recipe: 27 g agar, 33 g brown sugar, and 330 mL pure apple juice in 1000 mL diluted water) until experimental exposure to host larvae.

*D. melanogaster* lines *w^1118^* (BL#5905), *nub‐GAL4* (BL#86108), *UAS‐2×EGFP* (BL#6658), and *GMR‐GAL4* (BL#101859) were obtained from the Bloomington *Drosophila* Stock Center. The ubiquitous driver *da‐GAL4* (TB#00103) was obtained from the Tsing Hua Fly Center. *The D. melanogaster* lines *dilp8^MI00727^* (BL#33079), *UAS‐dilp8 RNAi1* (BL#80436), and *UAS‐dilp8 RNAi2* (VDRC#9420) were kindly provided by Dr. Junzheng Zhang (China Agricultural University, China). *UAS‐grim* (BCF#76), *D. simulans* (BCF#93), *D. yakuba* (BCF#94), *D. pseudoobscura* (BCF#95), and *D. virilis* (BCF#97) were obtained from the Core Facility of *Drosophila* Resource and Technology, Chinese Academy of Sciences. *D. sechellia* (k‐s10) and *D. erecta* (k‐s02) were obtained from the KYORIN‐Fly, Fly Stocks of Kyorin University. *D. suzukii* was collected in the above traps with parasitoid wasps.^[^ ^31^ ^]^ *D. santomea* was generously provided by Dr. Qi Zhou (Zhejiang University, China). All *Drosophila* species were reared on standard cornmeal/molasses/agar medium.^[^ ^33^ ^]^

### Parasitism Efficiency Assay

Two developmental stages of *D. melanogaster* hosts were used to assess the parasitism efficiency of parasitoid wasps: second‐instar larvae (younger hosts; 60 h after egg laying, 60 h AEL) and third‐instar larvae (older hosts; 96 h AEL). For non‐competition parasitism assays, either Aj or Ld was exposed to hosts at a 1:10 (wasp:host) ratio for varying durations. For competition parasitism assays, both parasitoid species were simultaneously introduced to hosts at a 1:1:10 (Aj:Ld:host) ratio and allowed to parasitize for varying durations. The wasp emergence rate, a key metric of parasitism efficiency, was calculated using the following formula: Wasp emergency rate = (number of emerged wasp adults/number of hosts) ×100%.

### Host‐Searching Behavioural Assay

*D. melanogaster* host larvae were placed in a 35 mm dish containing fly food. A mated 3‐day‐old female Aj or Ld was then introduced into the dish. For each wasp, the time required was recorded to successfully locate a host during its first three search attempts, with the average of the three measurements calculated as the individual's searching time. This assay was performed with 30 wasps per parasitoid species.

### Tissue Size Measurement

Imaginal discs (wing disc, eye‐antennal (EA) disc, leg disc, and haltere disc), and other tissues, including the central nervous system (CNS), salivary gland, prothoracic gland, and male/female gonads, were dissected from both parasitized and non‐parasitized *D. melanogaster* host larvae using a Leica stereomicroscope. The dissected tissues were fixed in 4% paraformaldehyde (in 1 × PBS) for 30 min at room temperature, followed by two washes in 1 × PBST (PBS containing 0.1% Triton X‐100 and 0.05% Tween 20) and one wash in 1 × PBS. The tissues were then mounted in ProLong Gold Antifade reagent with DAPI (Invitrogen, Cat# P36935). Images of the tissues were captured using a Zeiss LSM800 confocal microscope, and tissue size measurements were conducted using ImageJ software (v1.54g, National Institutes of Health).

### Apoptosis Analysis by TUNEL Staining

Apoptosis was analysed using the terminal deoxynucleotidyl transferase‐mediated dUTP nick end labelling (TUNEL) BrightRed Apoptosis Detection Kit (Vazyme, Cat# A113‐03) according to the manufacturer's protocol. Briefly, imaginal discs (wing, EA, leg, and haltere discs) were dissected from both parasitized and non‐parasitized *D. melanogaster* host larvae at 6 h post‐parasitization, fixed in 4% paraformaldehyde (in 1 × PBS) for 20 min at room temperature, and washed twice with 1 × PBS. The tissues were then permeabilized with Proteinase K (diluted in 1 × PBS) for 5 min, washed twice with 1 × PBS, followed by equilibration in TdT reaction buffer for 30 min at room temperature and incubation with a reaction mixture containing BrightRed labelling mix and TdT enzymes for 60 min at 37 °C in the dark. After two final washes with 1 × PBST (5 min each), the samples were mounted in ProLong Gold Antifade reagent with DAPI (Invitrogen, Cat# P36935) and immediately imaged using a Zeiss LSM800 confocal microscope.

### Quantitative Real‐Time PCR

Total RNA was extracted from either *D. melanogaster* host larval imaginal discs or whole larvae using the FastPure Cell/Tissue Total RNA Isolation Kit‐BOX2 (Vazyme, Cat# RC101‐01), followed by cDNA synthesis with HiScript III RT SuperMix for qPCR (Vazyme, Cat# R223‐01) according to the manufacturer's protocol. The resulting cDNA was used as a template for quantitative real‐time PCR (qRT‒PCR). qRT‒PCR was performed using ChamQ SYBR qPCR Master Mix (Vazyme, Cat# Q311‐02) on a QuantStudio3 Real‐Time PCR System (Thermo Fisher Scientific). Reactions were carried out for 30 s at 95 °C, followed by 40 cycles of three‐step PCR for 10 s at 95 °C, 20 s at 55 °C, and 20 s at 72 °C. Gene expression levels were normalized to *tubulin* (for Aj) or *actin 5C* (for *D*. m*elanogaster*), with relative quantification calculated using the 2^‐ΔΔCt^ method.^[^ ^51^ ^]^ Three biological replicates were analysed per gene, and the primer sequences are provided in Table S6 (Supporting Information).

### Venom and Ovarian Fluid Injection

Approximately 100 venom reservoirs and 100 pairs of ovaries were dissected from 3‐day‐old Aj females in 1 × PBS on an ice plate under a Leica stereomicroscope. The tissues were washed at least three times with 1 × PBS. Venom reservoirs were transferred to a sterile cell culture dish and punctured with fine forceps, and the released venom fluid was collected into a prechilled tube containing 60 µL of ice‐cold 1 × PBS. The protein concentration of the collected venom was quantified using a BCA protein assay kit (Invitrogen, Cat# 23225). The concentration was determined to be 2.1 µg µL^−1^. The ovaries were separately homogenized in 60 µL of 1 × PBS using sterile grinding beads. After centrifugation at 3000 × g for 1 min at 4 °C, the resulting supernatants (venom fluid and ovarian fluid) were stored at −80 °C for further use. For microinjection, ≈1/30 of the total venom extract (equivalent to a protein dose of ≈40 ng) or ovarian fluid from a single wasp was injected into third‐instar *D. melanogaster* larvae using an Eppendorf FemtoJet 4i device with an injection pressure of 900 hPa and an injection time of 0.10 s.

### Double‐Stranded RNA Preparation and Microinjection

Double‐stranded RNA (dsRNA) was synthesized using the T7 High Yield RNA Transcription Kit (Vazyme, Cat# TR101‐02). Gene‐specific primers with T7 promoters were designed to amplify 250–700 bp products of target VP genes (primer sequences and the corresponding genes are listed in Table S7, Supporting Information). A 438 bp green fluorescent protein (GFP) sequence served as the control (*dsGFP*). The concentration of purified dsRNA was quantified using a Nanodrop 2000 (Thermo Scientific, USA). Approximately 20 nL of dsRNA at a concentration of 5 µg µL^−1^ was injected into Aj prepupae using the Eppendorf FemtoJet 4i device with an injection pressure of 900 hPa and an injection time of 0.10 s. At least 100 Aj prepupae were injected for each dsRNA. After eclosion of the dsRNA‐treated parasitoids, they were used to parasitize the hosts. qRT‒PCR was used to identify the RNAi efficiency, and the results are listed in Figures S9 and S14 (Supporting Information).

### Chromosome Staining

Chromosome preparation for Aj karyotyping was performed using a modified protocol adapted from Imai et al.^[^ ^52^ ^]^ Briefly, ovaries were dissected from female Aj prepupae in sterile 1 × PBS, rinsed for 3–5 min in 1 × PBS, and incubated in 0.005% colchicine‐hypotonic solution (prepared in 1% sodium citrate) for 30 min before being transferred onto clean slides. The tissues were fixed with Fixative Solution A (anhydrous ethanol: glacial acetic acid: double‐distilled water at a 3:3:4 ratio by volume), gently disaggregated with forceps to ensure uniform chromosome spreading, and subsequently fixed with Fixative Solution B (anhydrous ethanol: glacial acetic acid at a 1:1 ratio by volume). Finally, the samples were mounted with ProLong Gold Antifade Mountant with DAPI (Invitrogen, Cat# P36935) and imaged using a Zeiss LSM 800 confocal microscope.

### Genome Sequencing

To generate a high‐quality chromosome‐level genome, Aj genomic DNA was subjected to comprehensive sequencing using PacBio long‐read and Hi‐C technologies. For PacBio sequencing, DNA was extracted from a pool of ≈1000 Aj adults to meet the requirements for library construction using the DNeasy Blood and Tissue Kit (Qiagen, Cat# 69506). DNA quality was checked using gel electrophoresis (0.8% agarose gel) and a High Sensitivity DNA Kit (Agilent, USA) in combination with a Bioanalyzer 2100 (Agilent, USA). A 20‐kb genomic library was constructed and sequenced on a PacBio Sequel platform by Berry Genomics Co., Ltd. (Beijing, China), generating a total of 32.4 Gb of raw reads (≈118 × genome coverage; Table S3, Supporting Information). The same DNA sample was additionally sequenced on the Illumina platform, producing 24.08 Gb of short reads (≈88 × coverage; Table S3, Supporting Information) for error correction.

Hi‐C libraries were prepared from a pool of 20 newly emerged Aj adults following the established protocol.^[^ ^53^ ^]^ The samples were fixed with 2% formaldehyde for 10 min at room temperature, after which 100 mm glycine solution was added to stop the cross‐linking reaction. The cross‐linked DNA was extracted and digested overnight with HindIII (NEB, Cat# N3012S). Biotin‐14‐dCTP17 was introduced during the sticky end repair process. The interacting DNA fragments were ligated using T4 DNA ligase to form chimeric junctions. Hi‐C libraries were sequenced on an Illumina HiSeq X Ten platform by Berry Genomics Co., Ltd. (Beijing, China), yielding 65.22 Gb of paired‐end reads (≈238 × genome coverage; Table S3, Supporting Information).

### Genome Assembly

PacBio reads were used to assemble contigs using Nextdenovo v2.4.0 with the following parameters: “read_type = clr read_cutoff = 2k genome_size = 900m seed_depth = 60 nextgraph_options = ‐a 1 ‐A”.^[^ ^54^ ^]^ The assembled contigs were subsequently corrected and polished using Illumina paired‐end reads via Nextpolish v1.3.1^[^ ^55^ ^]^ with the following parameters: “task = best rerun = 3 sgs_options = ‐max_depth 100 ‐bwa lgs_options = ‐min_read_len 1k ‐max_depth 100 lgs_minimap2_options = ‐x map‐pb”. The polished contigs underwent two rounds of redundancy removal using purge_dups v1.2.3 with default parameters,^[^ ^56^ ^]^ yielding a 297.77 Mb draft genome, with a contig N50 of 3.11 Mb across 433 contigs (Table S4, Supporting Information).

To achieve chromosome‐level assembly, the assembled contigs were clustered using Hi‐C contact information derived from Hi‐C sequencing reads. The Hi‐C contact signals were processed with Juicer v1.5.7^[^ ^57^, ^58^ ^]^ and subsequently analysed with 3D‐DNA v190716 using the parameters “‐q 1 –editor‐repeat‐coverage 2” for chromosome grouping.^[^ ^59^ ^]^ The chromosomal interaction matrix was visualized as a heatmap in Juicebox, displaying characteristic diagonal patterns of strong linkage based on interactions between valid mapped reads and genomic bins^[^ ^60^ ^]^ (Figure S7B, Supporting Information). The final chromosome‐level assembly had a size of 273.70 Mb with a scaffold N50 of 16.8 Mb and a GC content of 45.70% (Table S4, Supporting Information).

### Transcriptome Sequencing and Analysis

Since Aj was a thelytokous parthenogenetic species, all the transcriptomic samples were derived from female individuals. RNA sequencing of Aj developmental stages was performed to obtain comprehensive transcriptome profiles for each stage, including Egg, L1 (Days 1‐2 larvae; early larval stage), L2 (Day 3 larvae; middle larval stage), L3 (Days 4‐5 larvae; late larval stage), P1 (Days 1‐2 pupae; early pupal stage), P2 (Days 3–5 pupae; middle pupal stage), P3 (Days 6–8 pupae; late pupal stage), and AF (Days 3–5 adult females). In addition, the venom glands (VGs) of 3‐day‐old AF wasps were dissected in 1 × PBS solution on an ice plate under a stereoscope (Leica, Germany). Total RNA was isolated from each sample using FastPure Cell/Tissue Total RNA Isolation Kit‐BOX2 (Vazyme, Cat# RC101‐01). cDNA library construction and paired‐end RNA sequencing were conducted by Berry Genomics Co., Ltd. (Beijing, China) on an Illumina NovaSeq 6000 platform (Illumina, USA). The statistics of the transcriptome sequencing data are listed in Table S8 (Supporting Information). Gene expression values across developmental stages or tissues were quantified as transcripts per million (TPM) values via salmon v0.12.0,^[^ ^61^ ^]^ with the parameters “quant ‐l A”, and gene expression visualization was performed using the R package “pheatmap”.

Full‐length transcript sequencing was also performed using the PacBio sequencing system for genome annotation. A total of 9.83 Gb of transcriptome data were generated from libraries with 1–10 kb inserts, representing pooled mRNAs from all Aj developmental stages (Table S8, Supporting Information). The raw reads were processed with isoseq v3.2.2 and aligned to the reference genome using Minimap v2.17 with the parameters “‐ax splice ‐uf ‐secondary = no C5”.^[^ ^62^ ^]^

To compare the RNA expression profiles of host imaginal discs following parasitism, wing and EA discs were collected from both non‐parasitized control larvae and Aj‐parasitized third‐instar host larvae at 6 h post‐parasitization for transcriptome analysis. RNA extraction and sequencing were performed using the protocols described above. Gene read numbers were quantified using salmon v0.12.0 with the parameters “quant ‐l A”.^[^ ^61^ ^]^ Differentially expressed genes were identified using the R package edgeR with Benjamini‒Hochberg false discovery rate (FDR) correction (FDR < 0.01; |log_2_(fold change)| >1). Data visualization was performed using the R package ggplot2.

### Genome Annotation

The repeat contents in the genome were annotated using the RepeatMasker pipeline (https://www.repeatmasker.org/RepeatMasker/). First, a species‐specific repeat library for Aj was constructed using RepeatModeler v2.0.2. RepeatMasker v4.1.1 was subsequently used to mask the repeat contents across the whole genome using both the Aj‐specific library and the Dfam v3.2 database.^[^ ^63^ ^]^

Protein‐coding genes were predicted based on repeat‐masked genome. Multiple gene prediction approaches were used to generate several gene sets, including (1) BRAKER v2.1.5 was used to generate two gene sets, one based on transcriptome‐based hints and the other on related protein‐based hints;^[^ ^64^, ^65^, ^66^ ^]^ (2) MAKER v2.31.10 was used to generate an integrated gene set by combining SNAP v2006‐07‐28 and Augustus v3.3.2 predictions with evidence from related proteins and full‐length transcripts;^[^ ^67^, ^68^, ^69^ ^]^ (3) StringTie v2.0 was used to combine all Illumina‐based transcriptome data to generate a merged transcript set using default parameters;^[^ ^70^ ^]^ (4) the python module “collapse_isoforms_by_sam” of TOFU was used to generate a full‐length read‐based transcript set, with parameters of “‐dun‐merge‐5‐shorter ‐c 0.9 ‐i 0.9”.^[^ ^71^ ^]^ All of the above independent gene sets were subjected to pairwise comparisons at both the transcript level and the exon level, with consensus predictions retained as priorities and multi‐supported predictions secondarily, while single‐source predictions were excluded. The predicted genes were then annotated with functions based on both BLASTP against a local InterProScan search (v5.56–89.0) for domains.^[^ ^72^ ^]^ The final gene set comprised 13931 protein‐coding genes, with a mean gene length of 1465.3 bp (Table S4, Supporting Information).

### Venom Proteome Analysis via Liquid Chromatography–Tandem Mass Spectrometry (LC‒MS/MS)

The venom reservoirs from 3‐day‐old Aj female wasps were dissected in 1 × PBS solution on an ice plate under a stereoscope (Leica, Germany). Venom fluid collected from ≈100 venom reservoirs was dissolved in 100 µL of SDT lysis buffer (4% SDS, 100 mm Tris‐HCl, pH 7.6). The venom sample was boiled for 15 min and centrifuged at 13 000 × g at 4 °C for 40 min. The venom protein concentration in the supernatant was quantified with a BCA protein assay kit (Invitrogen, Cat# 23225). A 20 µg aliquot of venom protein was digested into peptides with trypsin. The resulting peptides were desalted using C18 cartridges (Sigma, USA) and reconstituted in 40 µL of 0.1% (v/v) formic acid.

LC‒MS/MS analysis was performed using a timsTOF Pro mass spectrometer (Bruker, Germany) coupled to a NanoElute system (Bruker Daltonics, Germany). Peptides were loaded onto a custom C18 reversed‐phase analytical column (25 cm length, 75 µm inner diameter) with buffer A (0.1% formic acid) and separated using a linear gradient of buffer B (99.9% acetonitrile/0.1% formic acid) at a 300 nL min^−1^ flow rate. The mass spectrometer was operated in positive ion mode, and ion mobility MS spectra were collected across a mass range of m/z 100–1700 and an ion mobility range (1/k0) of 0.75–1.35. Subsequent analysis included 10 cycles of PASEF MS/MS, targeting an intensity of 1.5k with a threshold of 2500. Active exclusion was implemented with a release time of 0.4 min. The raw data were analysed using the MASCOT engine v2.2 (Matrix Science, UK) against the Aj protein sequence database.

### Identification of Parasitoid Venom Protein (VP) Genes

Aj VP genes were identified through combined transcriptomic and proteomic evidence. Genes with transcripts per million (TPM) values higher than 6.91 (N99 value) in the venom were defined as venom gland‐expressed genes. Among them, the genes that could be fully aligned to at least two proteomic peptides, plus the presence of signal peptides, were defined as VP genes. Functional enrichment analysis of InterPro terms was performed using a hypergeometric test with Benjamini‒Hochberg FDR correction. To support the annotation of VP genes, all predicted protein sequences were searched against the NCBI non‐redundant (NR) database and a custom database of venom proteins from *M. demolitor*, *N. vitripennis*, and *N. giraulti* by BLASTP v2.12.0 with an E‐value cutoff of 1e−5. The top hits for each gene were manually curated to generate functional descriptions (Table S5, Supporting Information).

VP genes from additional parasitoid species (*M. demolitor*, *T. drosophilae*, *L. drosophilae*, *N. vitripennis*, and *N. giraulti*) were identified using the same method, with proteomic data obtained from the ProteomeXchange Consortium (*M. demolitor*, PXD007905; *T. drosophilae*, PXD038251; *L. drosophilae*, PXD054736) and FigShare (*N. vitripennis* and *N. giraulti*, (https://doi.org/10.6084/m9.figshare.4731850), whereas venom gland transcriptomic data were acquired from NCBI (*M. demolitor*, SRR955397; *T. drosophilae*, SRR23044234; *L. drosophilae*, SRR30310320; *N. vitripennis*, SRR4419177; *N. giraulti*, SRR4419178).

### Evolutionary Analyses

The DUF4803‐domain (IPR032062) genes were systematically analysed across gene sets from 16 parasitoid species with high‐quality genomes using InterProScan v5.56–89.0.^[^ ^72^ ^]^ Genomic data for *M. demolitor* (IBG_00552), *Diachasmimorpha longicaudata* (IBG_03266), *Microctonus hyperodae* (IBG_03635), *Microctonus aethiopoides* (IBG_03634), *Meteorus cinctellus* (IBG_03629), *M. brassicae* (IBG_01313), *Cotesia congregata* (IBG_00207), *N. vitripennis* (IBG_00564), *Pteromalus puparum* (IBG_00672), *N. giraulti* (IBG_00562), and *T. drosophilae* (IBG_03945) were acquired from InsectBase 2.0 (http://v2.insect‐genome.com/), while genomic sequences for *L. drosophilae* (GCA_032873175.1), *C. vestalis* (LQNH00000000.1), *Aphidius gifuensis* (GCA_014905175.1), and *Fopius arisanus* (GCF_000806365.1) were obtained from the NCBI GenBank.

The DUF4803‐domain genes from six parasitoid wasp species (*Aj*, *M. demolitor*, *T. drosophilae*, *L. drosophilae*, *N. vitripennis*, and *N. giraulti*), which all possess high‐quality genomes, venom gland transcriptomes, and venom proteomic data, were aligned using MAFFT v7.487.^[^ ^73^ ^]^ Conserved regions were then extracted using trimAl v1.4.rev22 with the ‐auto parameter.^[^ ^74^ ^]^ A maximum likelihood phylogenetic tree was constructed with IQ‐TREE v2.1.3^[^ ^75^ ^]^ and visualized using iTOL (https://itol.embl.de/).

### Statistical Analysis

All the statistical analyses were performed using GraphPad Prism v10.1.2 (GraphPad Software, USA) and SPSS 27 (IBM, USA). The normal distribution of all the data was checked using the Shapiro‒Wilk test, and the homogeneity of variance of all the data was checked via the Fligner‒Killeen test. For comparisons between two groups, two‐tailed unpaired Student's *t* test was used when parametric assumptions and homogeneity of variances were met (Figures 2A, 4J; Figures S3 and S14, Supporting Information), Welch's *t* test was used when parametric assumptions were met but heterogeneity of variances was observed (Figures 2A,4D; Figures S5B,C,S9,S14 and S15, Supporting Information), and the Mann‒Whitney U test was used for nonparametric data (Figure 2A; Figures S1 and S3, Supporting Information). For multiple group comparisons, one‐way ANOVA with Sidak's multiple comparisons test (Figure 2, 3,4G,5C; Figures S11 and S16, Supporting Information) and two‐way ANOVA with Sidak's multiple comparisons test (Figure 1C,4K; Figure S2, Supporting Information) were used when parametric assumptions and homogeneity of variances were met. Details of the statistical analysis were provided in the figure legends. The data represent the mean ± standard error of the mean (SEM). Different letters indicate statistically significant differences (*p* < 0.05). For other tests, significance values were indicated as ns: not significant; ^*^, *p* < 0.05; ^**^, *p* < 0.01; ^***^, *p* < 0.001.

## Conflict of Interest

The authors declare no conflict of interest.

## Author Contributions

J.Z. and Z.D. contributed equally to this work. Conceived and designed the experiments: J.H. and J.C. Performed the experiments: J.Z., Y.S., J.S., T.F., W.S., and Z.W. Analyzed the data: J.Z., Z.D., and Z.X. Contributed materials/analysis tools: J.C., J.Z., Z.D., Q.Z., and Y.W. Wrote the paper: J.H., J.C., J.Z., and Z.D.

## Supporting information

Supporting Information

Supporting Information

Supporting Information

Supporting Information

Supporting Information

Supporting Information

## Data Availability

The genomic data generated in this study have been deposited in NCBI GenBank under BioProject accession numbers PRJNA1207244, PRJNA1207610, PRJNA1241485, and PRJNA1207731. The transcriptomic data are available in the NCBI GenBank under BioProject accession numbers PRJNA1207143, PRJNA1206545, and PRJNA1208136. The MS proteome data for Aj venom have been deposited in the ProteomeXchange Consortium with the dataset identifier PXD059653. The coding sequences of 5 VID genes have been deposited in NCBI GenBank with the accession numbers PQ849756 (*VID‐1*), PQ849757 (*VID‐2*), PQ849758 (*VID‐3*), PQ867220 (*VID‐4*), and PQ867221 (*VID‐5*).
