# Supplementary material for: A Set of Novel Venom Proteins Enables Parasitoid Wasps to Exploit Older Hosts and Coexist with Competitors
Source: Adv Sci (Weinh). 2025 Nov 5;13(4):e12654. doi: 10.1002/advs.202512654 (PMC12822402; doi:10.1002/advs.202512654)
Supplement: Supplementary file 1 — Supporting Information [file ADVS-13-e12654-s006.docx]

Supporting Information

**A set of novel venom proteins enables parasitoid wasps to exploit older hosts and coexist with competitors**

*Junwei Zhang, Zhi Dong, Yifeng Sheng, Jieyu Shan, Ting Feng, Wenqi Shi, Zixuan Xu, Zeying Wang, Qichao Zhang, Ying Wang, Jianhua Huang* and Jiani Chen**


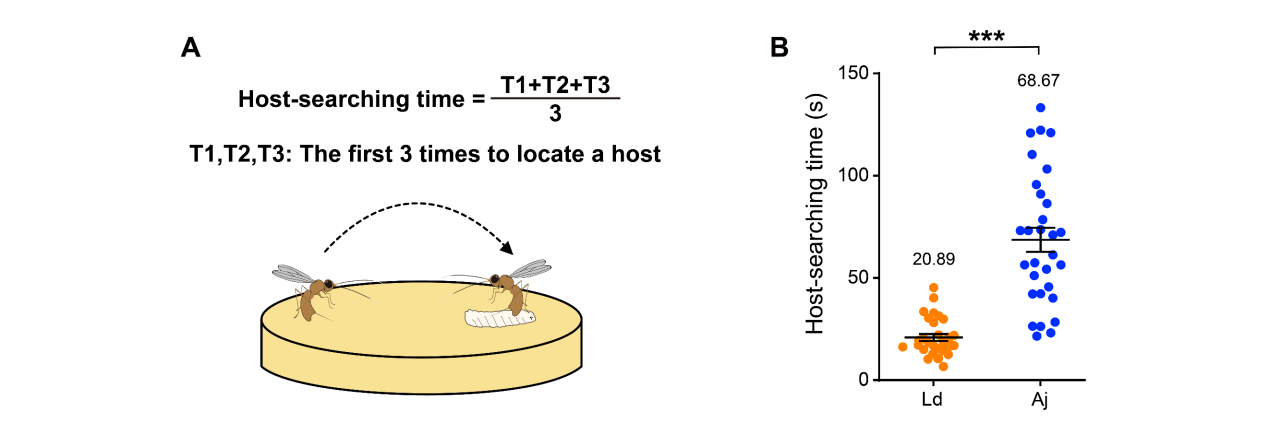
**Figure S1.** **Ld exhibits superior host-searching efficiency compared with Aj.**

**(A)** Schematic diagram of the host-searching time for Aj or Ld females to locate their 2nd-instar host larvae on fly food medium. The host-searching time was defined as the average of the first three successful host location events by an individual parasitoid wasp. **(B)** Host-searching time of Aj or Ld females. Each plot indicates the host-searching time for an individual female wasp. Thirty female wasps were used for each species. Data are presented as the mean ± SEM, with the average host-searching time shown for each species. Significance was analysed by the Mann‒Whitney U test (***, *p* < 0.001).
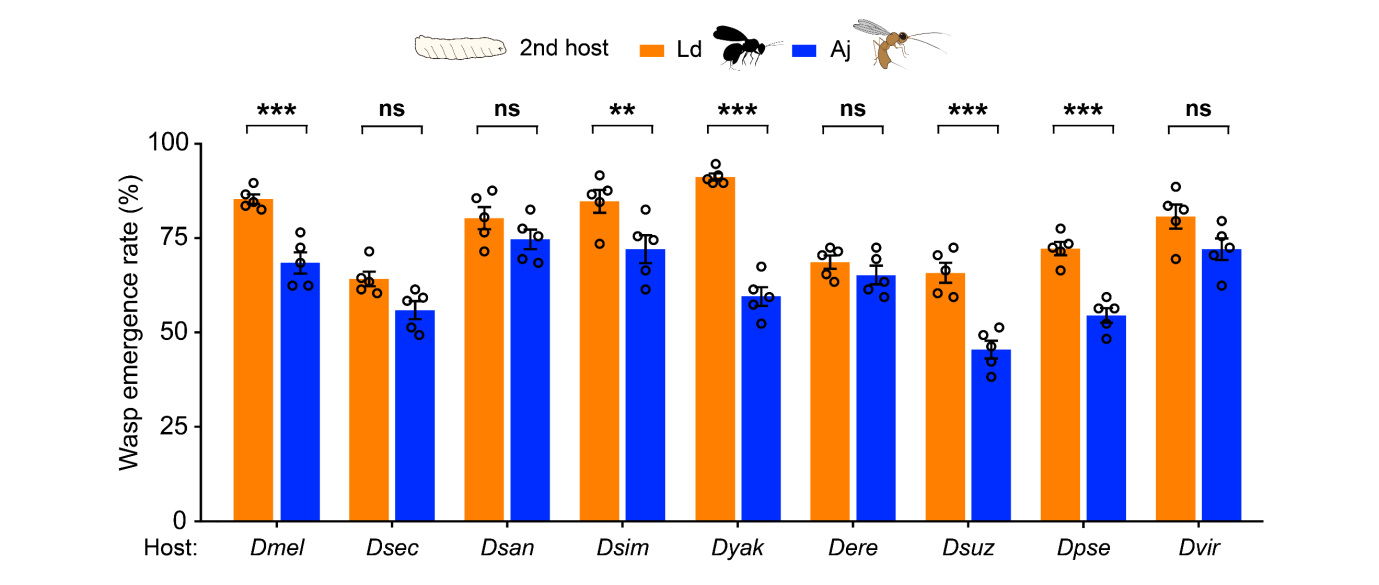
**Figure S2.** **Wasp emergence rates of Aj and Ld in different** ***Drosophila* host species.**

Comparison of wasp emergence rates in 9 *Drosophila* host species parasitized by Aj and Ld. Specifically, 3-day-old adult females of either Aj or Ld were allowed to parasitize 2nd-instar host larvae of different *Drosophila* species at a wasp:host ratio of 1:10. The exposure time was set at 20 min for Ld and 120 min for Aj, as these durations ensured high parasitization efficiency for both species (see Figure 1). Five biological replicates were performed. Data are presented as the mean ± SEM. Significance was analysed by two-way ANOVA with Sidak’s multiple comparisons test (ns, not significant; **, *p* < 0.01; ***, *p* < 0.001). *Dmel*, *D. melanogaster*; *Dsec*, *D. sechellia*; *Dsan*, *D. santomea*; *Dsim*, *D. simulans*; *Dyak*, *D. yakuba*; *Dere*, *D. erecta*; *Dsuz*, *D. suzukii*; *Dpse*, *D. pseudoobscura*; *Dvir*, *D. virilis*.
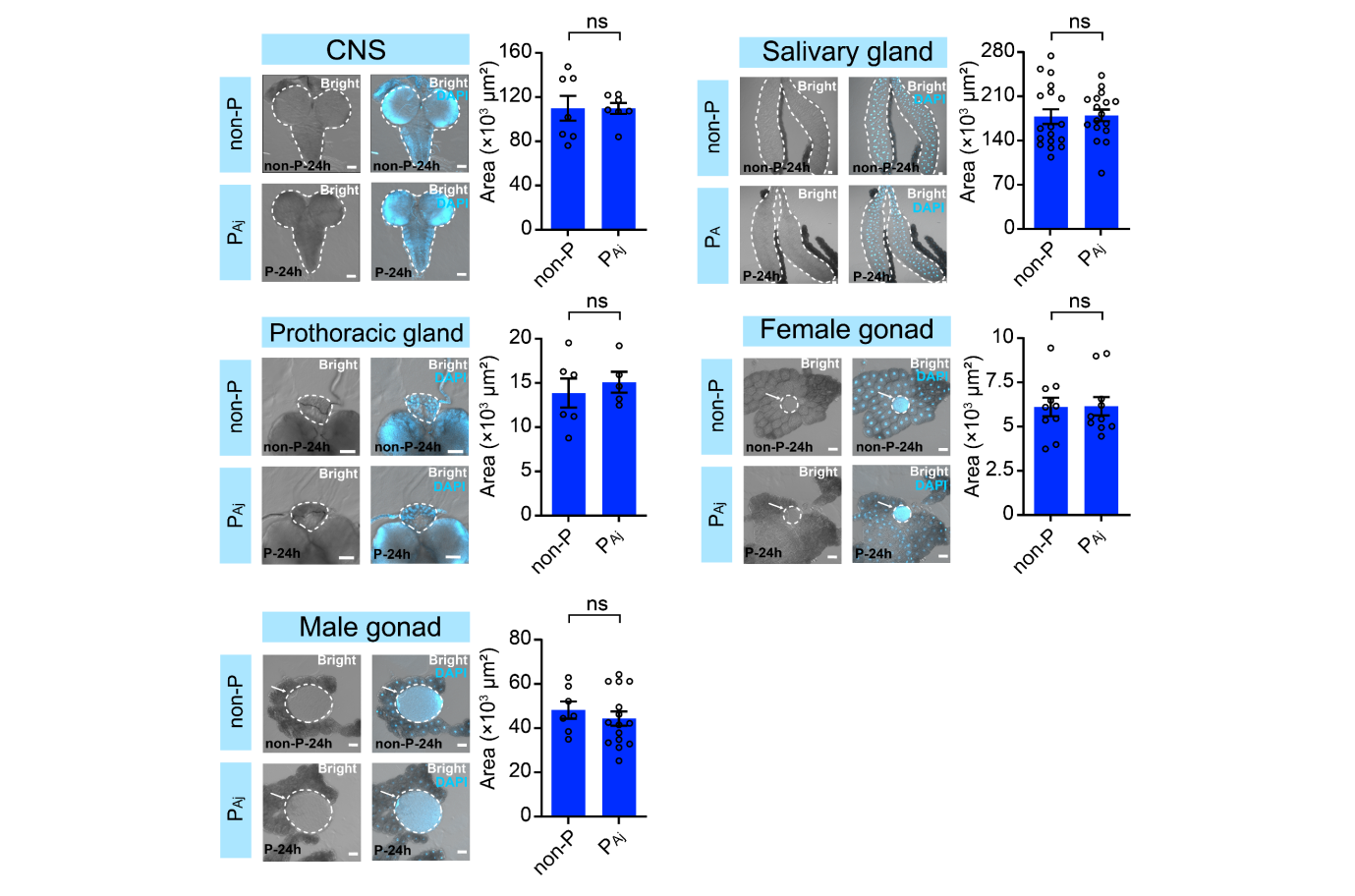
**Figure S3. Aj parasitization does not affect the size of non-imaginal disc host tissues.**

Representative fluorescence images of the central nervous system (CNS), salivary gland, prothoracic gland, female gonad and male gonad from non-parasitized (non-P) and Aj-parasitized (P_Aj_) 3rd-instar host larvae at 24 h post-parasitization (P-24 h). The bright field image is shown in the Bright channel, while the nuclei of the non-imaginal disc host tissues are labelled with DAPI (blue). The dashed lines mark the outlines of the non-imaginal disc host tissues. At least 5 tissues of each type were analysed for size. Data are presented as the mean ± SEM. Statistical analysis was performed using two-tailed unpaired Student’s t test when parametric assumptions and homogeneity of variances were met, and the Mann‒Whitney U test was used to determine significance when experiments required nonparametric statistical tests (ns: not significant). Scale bar: 50 μm.


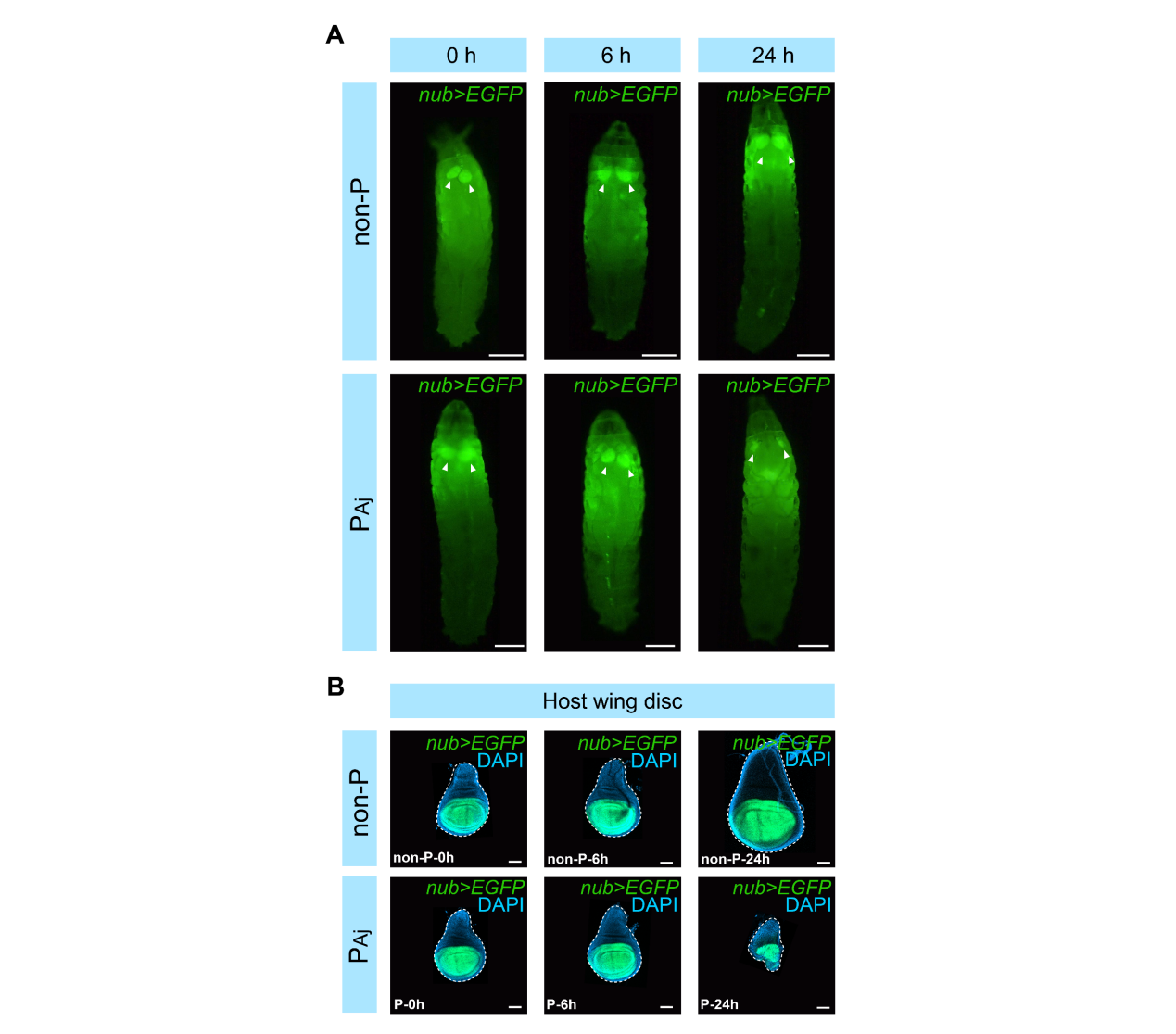
**Figure S4. Aj parasitization significantly reduces the size of the host wing imaginal discs.**

**(A)** Wing discs of non-parasitized (non-P) and Aj-parasitized (P_Aj_) 3rd-instar host larvae at 0 h, 6 h, and 24 h post-parasitization. While the size of wing discs was significantly reduced at 24 h post-parasitization, no obvious morphological changes were observed at 0 h or 6 h post-parasitization in the P_Aj_ group compared with the non-P group. At least 50 *Drosophila* 3rd-instar larvae from each group were examined at each time point. The nuclei were stained with DAPI (blue), and the wing discs were labelled with *nub>EGFP* (green). The dashed lines mark the outlines of the wing discs. Scale bars: 500 μm. **(B)** Wing discs from the relative hosts in **(A)**. The nuclei were labelled with DAPI (blue). The dashed lines mark the outlines of the wing discs. Scale bars: 50 μm.


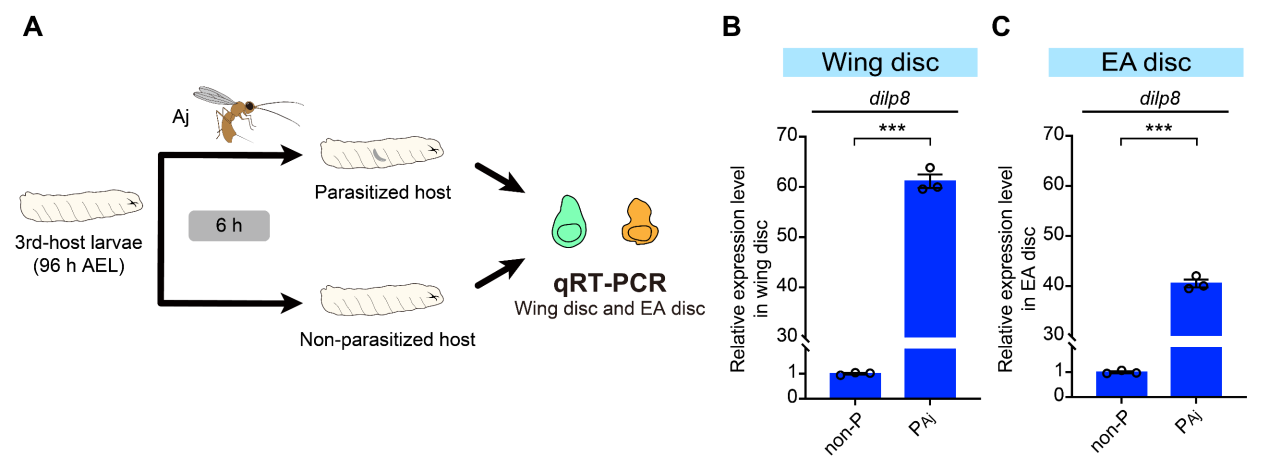
**Figure S5. Relative expression level of *dilp8* in host imaginal discs after Aj parasitization.**

**(A)** Scheme for collecting non-parasitized and parasitized host imaginal disc samples for qRT‒PCR. **(B**-**C)** Relative mRNA level of *dilp8* in wing discs **(B)** and EA discs **(C)** at 6 h post Aj parasitization. Three biological replicates were performed. Data are presented as the mean ± SEM. Significance was determined by Welch’s t test (***, *p <* 0.001).


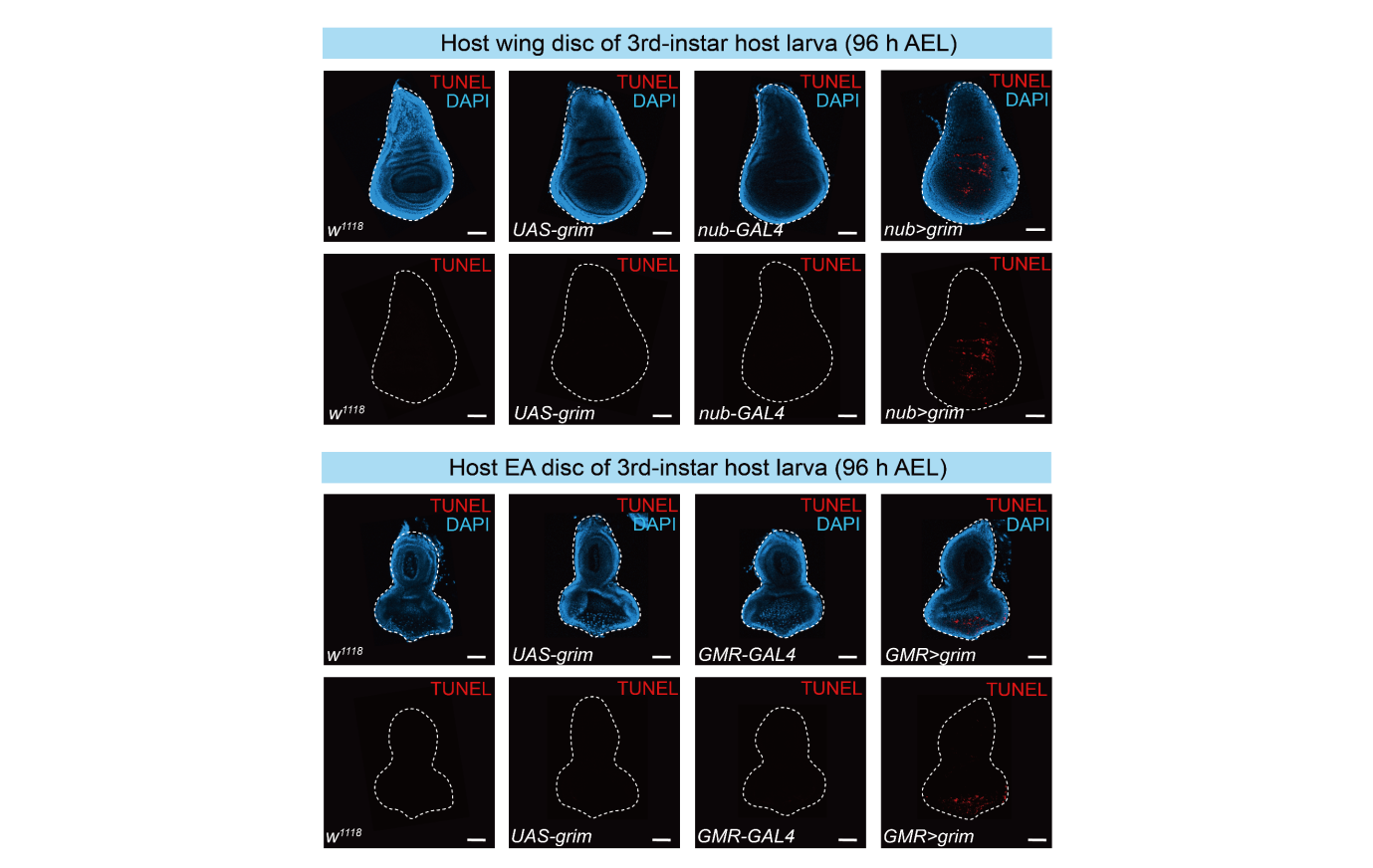
**Figure S6. Overexpression of *grim* in host imaginal discs induces ectopic cell apoptosis.**

Compared with the control wing discs (upper panel, *w^1118^*, *UAS-grim* and *nub-GAL4*) and EA discs (lower panel, *w^1118^*, *UAS-grim* and *GMR-GAL4*), obvious apoptotic signals were present in the *nub-GAL4>UAS-grim* (*nub>grim*) wing discs and *GMR-GAL4>UAS-grim* (*GMR>grim*) EA discs. The wing and EA discs were dissected from 3rd-instar host larvae (96 h AEL). At least 30 imaginal discs of each genotype were examined. Cell apoptosis was visualized with TUNEL (red), and the nuclei were labelled with DAPI (blue). The dashed lines mark the outlines of the imaginal discs. Scale bars: 50 μm.


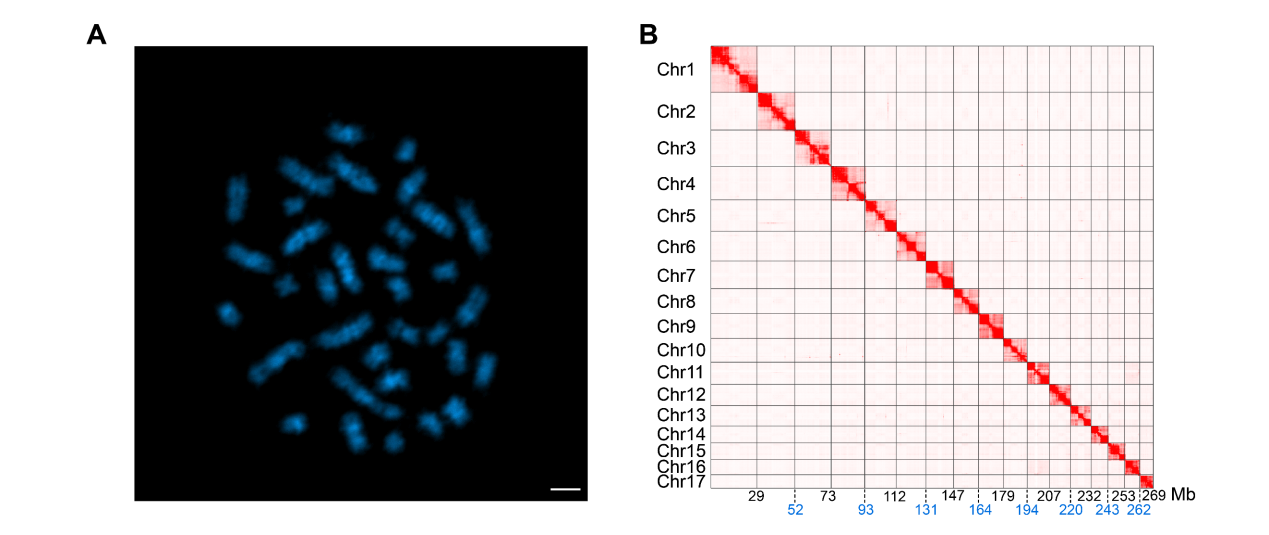
**Figure S7. Characteristics of the Aj genome.**

**(A)** Chromosome staining karyotypes of female Aj (2n=34). Scale bars: 2 μm. **(B)** Heatmap of Hi-C all-by-all interactions among the 17 chromosomes of Aj. The heatmap colours ranging from white to red indicate the frequency of Hi-C interaction links from low to high. The x-axis refers to each chromosome with an assembly scaffold length. The y-axis refers to each chromosome (Chr1-Chr17).


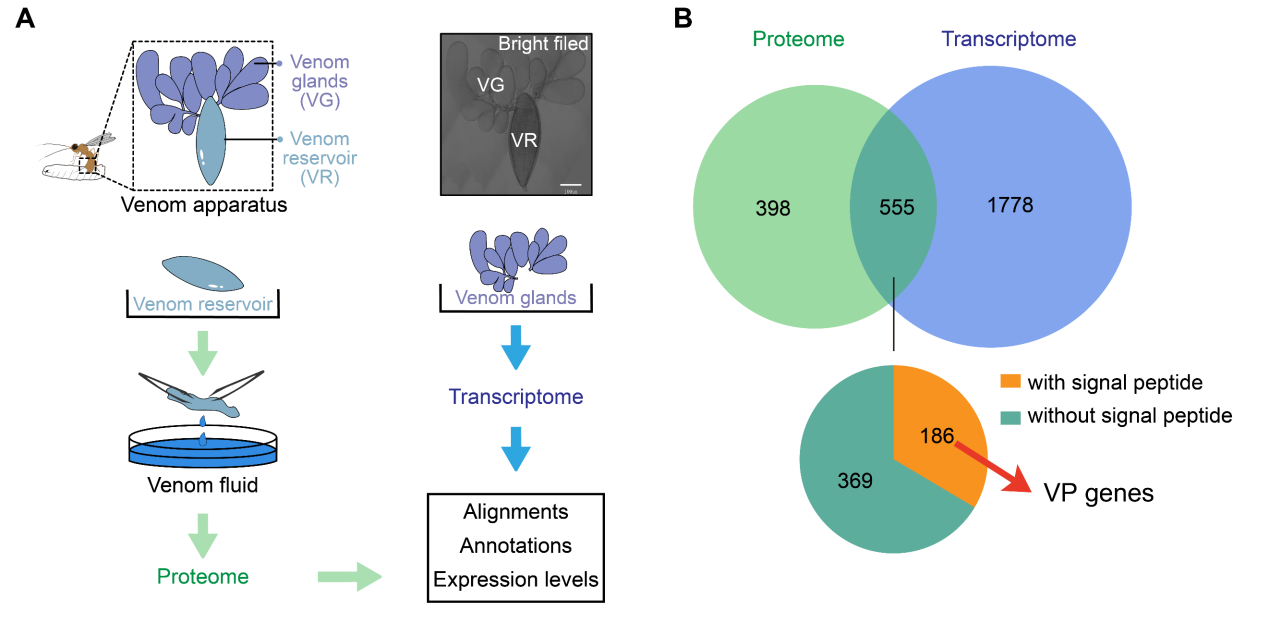
**Figure S8. Identification of Aj venom protein genes.**

**(A)** Schematic diagram of the identification of Aj venom protein (VP) genes. **(B)** Venn diagram illustrating the overlap between the venom proteome and the venom gland transcriptome. Genes with transcripts per million (TPM) values higher than 6.91 (N99 value) in the venom gland transcriptome were defined as venom gland-expressed genes. The genes that could be fully aligned to at least two proteomic peptides plus the presence of signal peptides were defined as Aj VP genes.


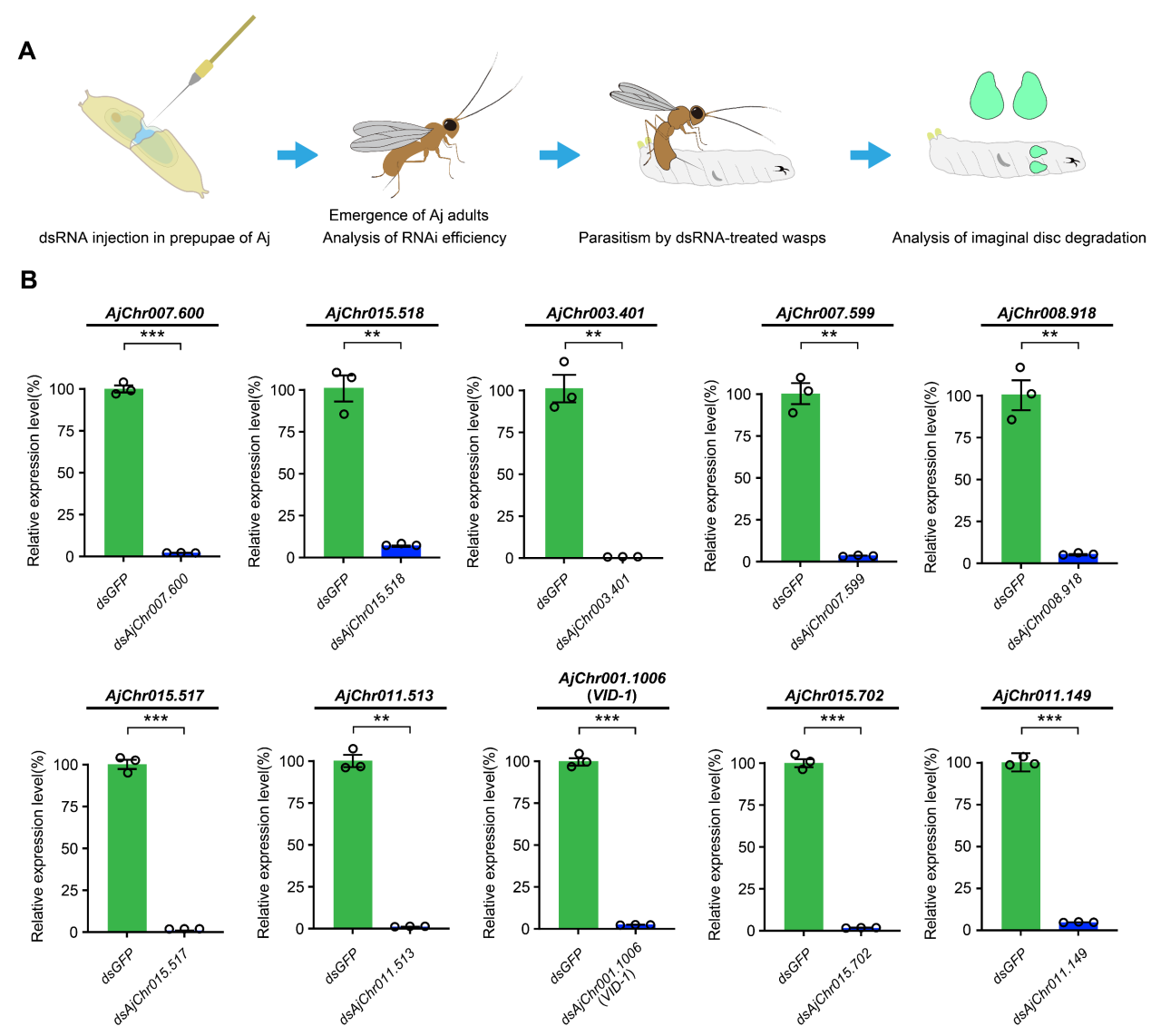
**Figure S9. RNAi efficiency of the top 10 highly expressed** **Aj VP genes.**

**(A)** Schematic diagram of the analysis of the role of candidate genes in host imaginal disc degradation by RNA interference (RNAi). **(B)** Relative mRNA levels of the top 10 highly expressed genes in Aj venom glands after RNAi treatment. Three replicates were performed. Data are presented as the mean ± SEM. Significance was determined by Welch’s t test (**, *p <* 0.01; ***, *p <* 0.001).


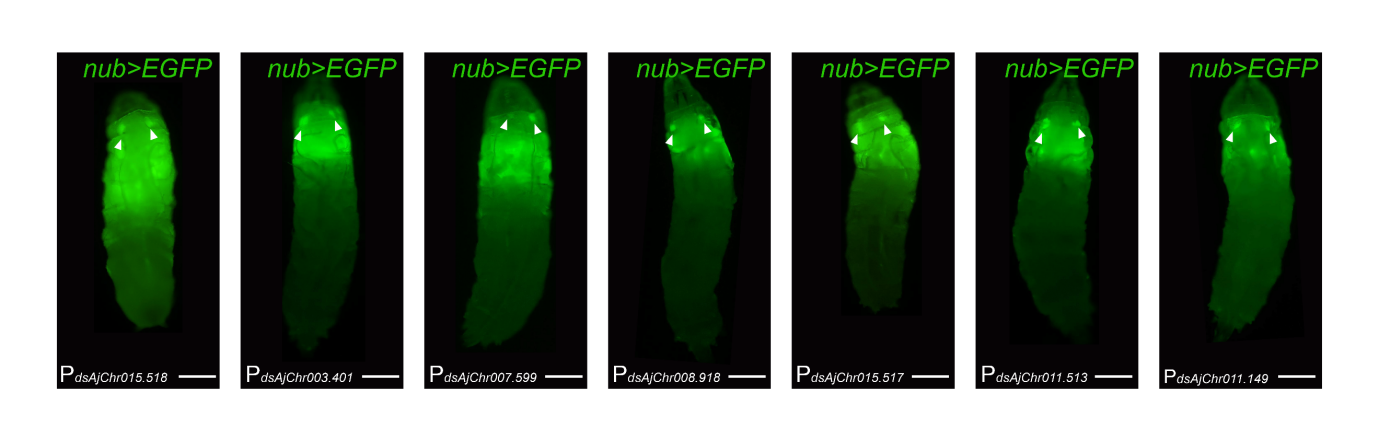
**Figure S10. Other highly expressed VP genes are not associated with host disc degradation.**

Host wing discs (indicated by white arrowheads) labelled with *nub>EGFP* (green) were examined for apoptosis-mediated degradation in Aj-parasitized 3rd-instar larvae at 24 h post-parasitization. The parasitized groups included 7 *dsRNA*-treated conditions: P*_dsAjChr015.518_*, P*_dsAjChr003.401_*, P*_dsAjChr007.599_*, P*_dsAjChr008.918_*, P*_dsAjChr015.517_*, P*_dsAjChr0011.513_*, and P*_dsAjChr011.149_*. Sixty *Drosophila* larvae from each treatment group were examined. Scale bars: 500 μm.


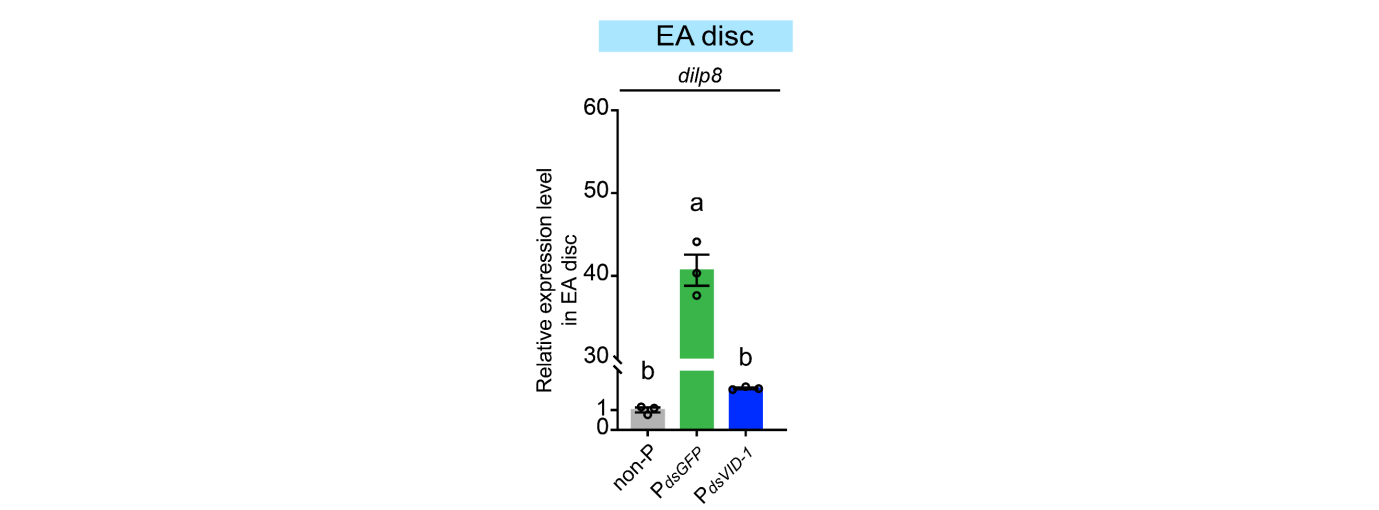
**Figure S11. Knockdown of *VID-1* suppresses *dilp8* induction in the EA discs of parasitized hosts.**

Relative expression level of *dilp8* in EA discs of P*_dsVID-1_* 3rd-instar host larvae at 6 h post-parasitization compared with P*_dsGFP_* and non-P controls. Three biological replicates were performed. Data are presented as the mean ± SEM. Significance was determined by one-way ANOVA with Sidak’s multiple comparisons test. Different letters indicate statistically significant differences (*p* < 0.05).


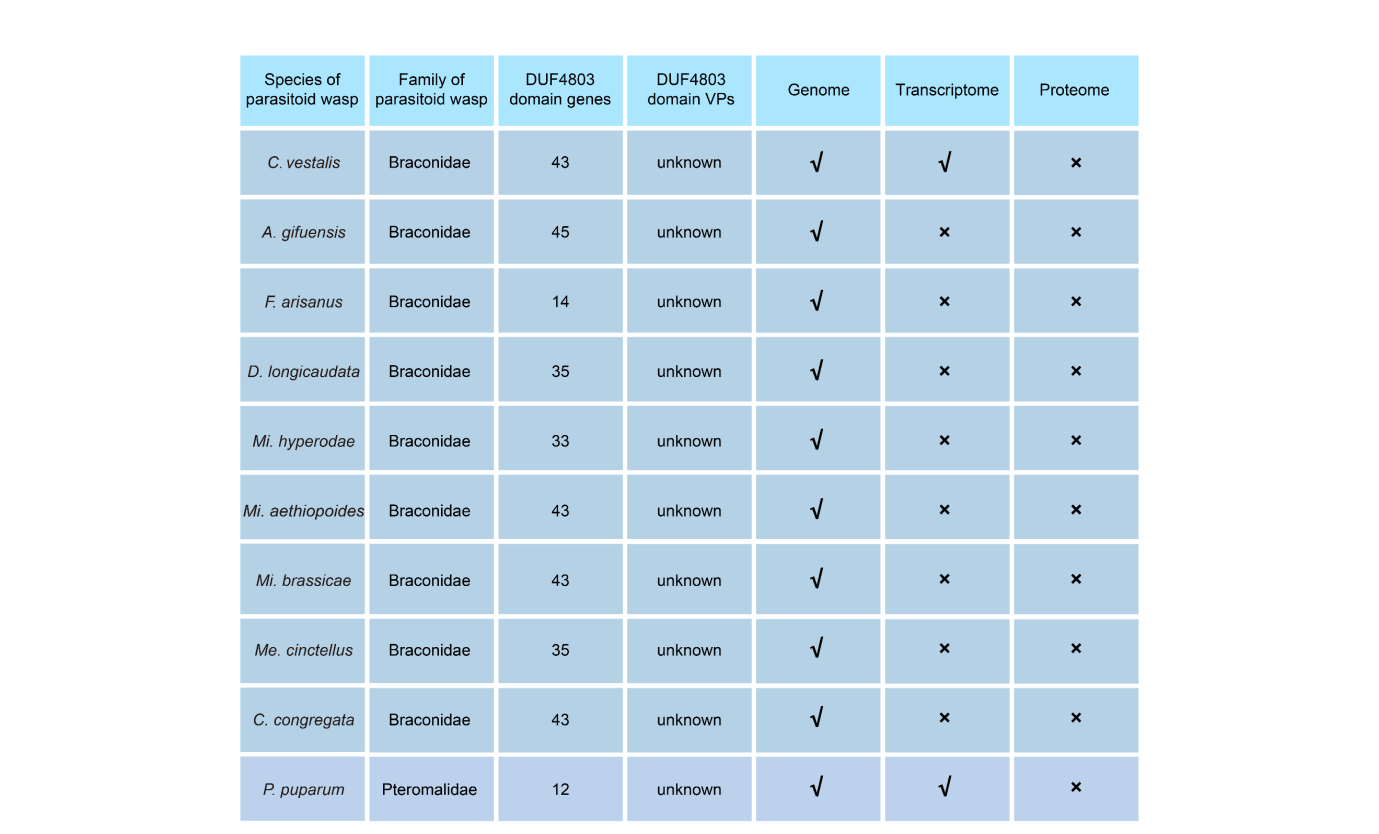
**Figure S12. Expansion information of DUF4803-domain genes in parasitoid species.** The numbers of DUF4803-domain genes and DUF4803-domain VP genes are shown in the table. These ten parasitoid species have high-quality genomes but lack venom gland transcriptome or proteomic data. The symbol “√” represents presence, and the symbol “×” represents absence. *C. vestalis*, *Cotesia vestalis*; *A. gifuensis*, *Aphidius gifuensis*; *F. arisanus*, *Fopius arisanus*; *D. longicaudata*, *Diachasmimorpha longicaudata*; *Mi. hyperodae*, *Microctonus hyperodae*; *Mi. aethiopoides*, *Microctonus aethiopoides*; *Mi. brassicae*, *Microctonus brassicae*; *Me. cinctellus*, *Meteorus cinctellus*; *C. congregata*, *Cotesia congregata*; *P. puparium*, *Pteromalus puparum*.


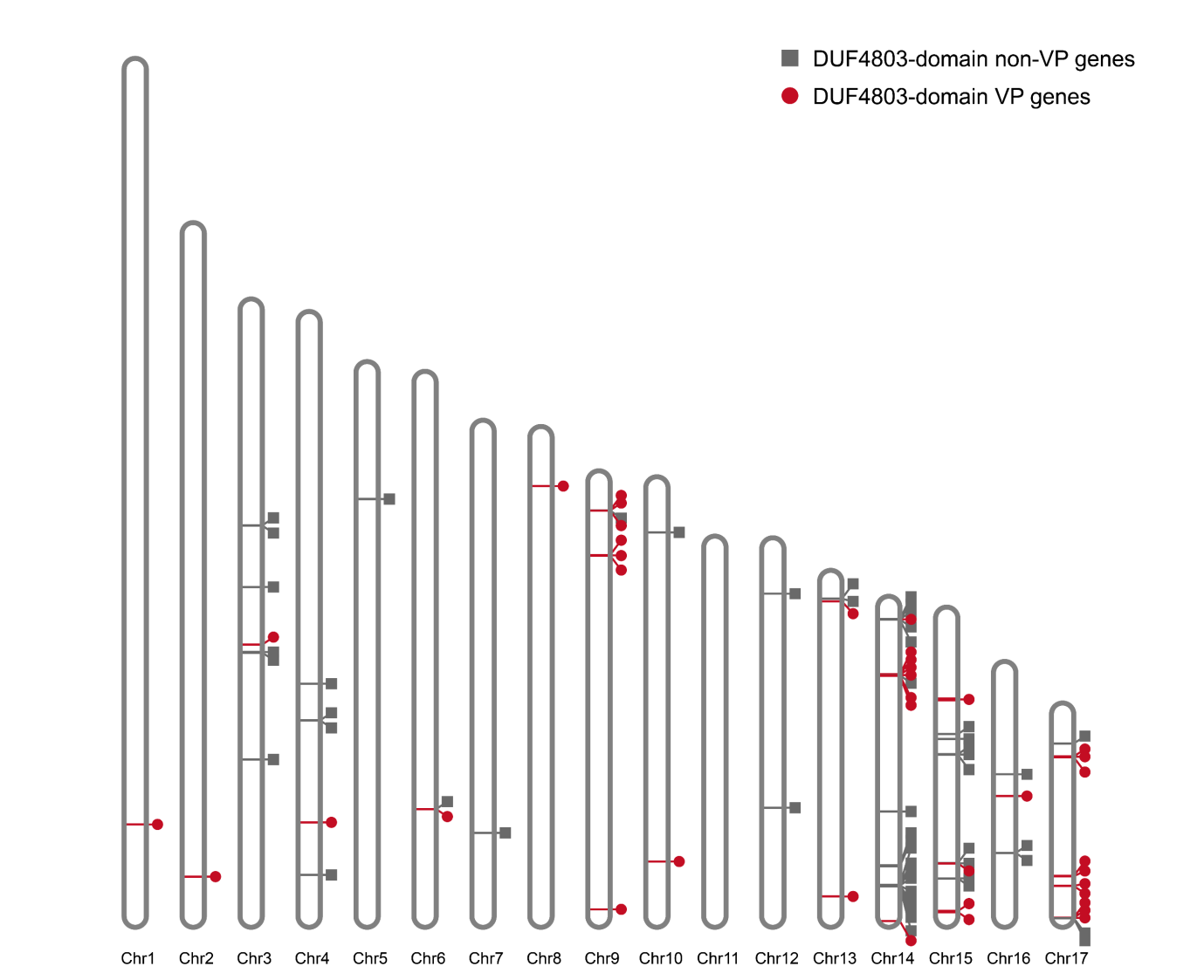
**Figure S13. Distribution of DUF4803-domain genes on Aj chromosomes.**

Genomic locations of characterized DUF4803-domain genes on seventeen Aj chromosomes. The red points represent the venom protein genes within these DUF4803-domain genes, and the gray squares represent the non-venom protein genes in this gene set.


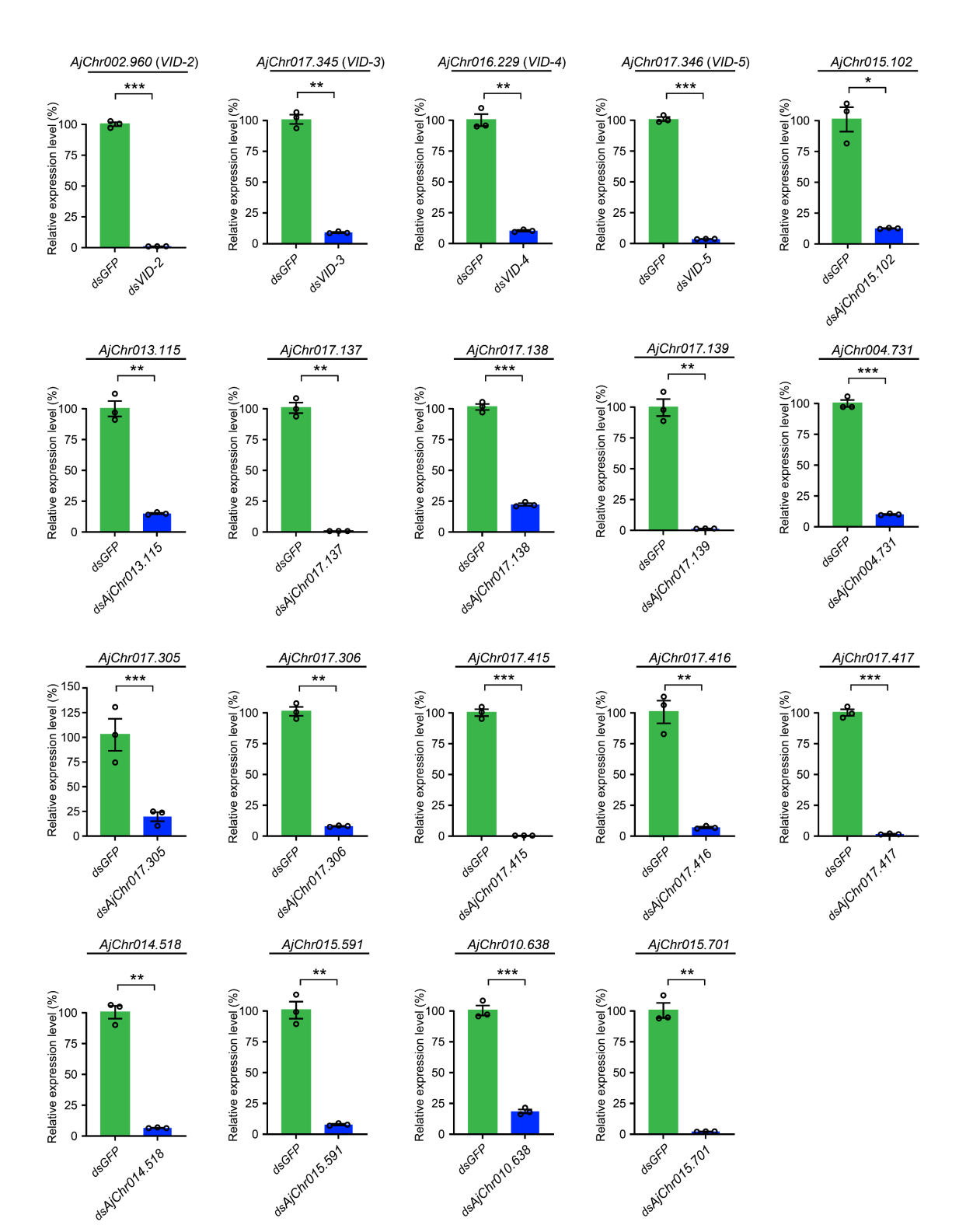
**Figure S14. RNAi efficiency of DUF4803-domain VP genes in clade V.**

Relative mRNA levels of DUF4803-domain VP genes in clade V after RNAi treatments, except for *AjChr001.1006* (*VID-1*) and *AjChr015.702*, which are shown in Figure S9. Three replicates were performed. Data are presented as the mean ± SEM. Statistical analysis was performed using two-tailed unpaired Student’s t test when parametric assumptions and homogeneity of variances were met, and Welch’s t test was used to determine significance when parametric assumptions were met but heterogeneity of variances was observed. (*, *p <* 0.05; **, *p <* 0.01; ***, *p <* 0.001).


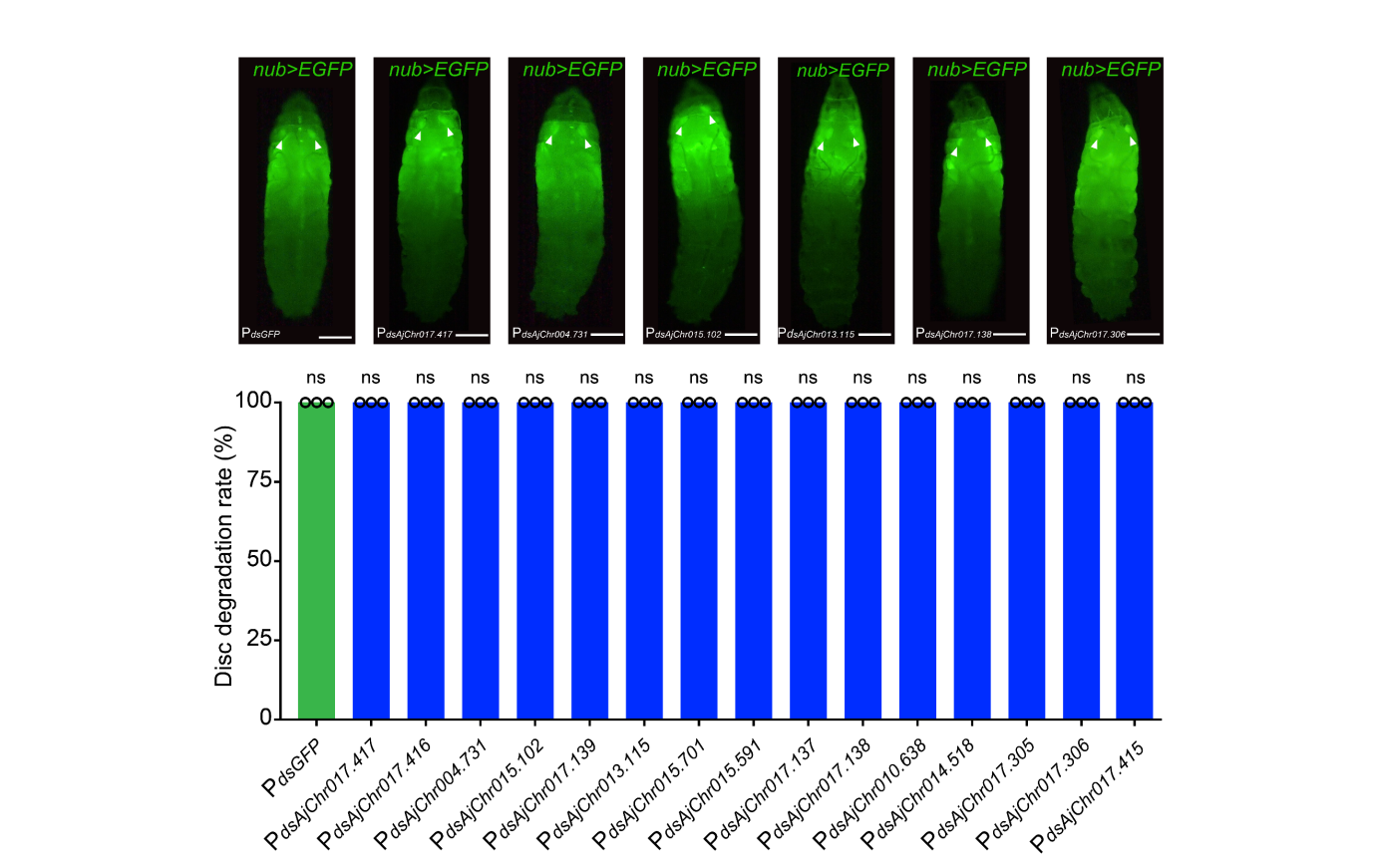
**Figure S15. Non-*VID* DUF4803-domain VP genes in clade V are not associated with host disc degradation.**

Disc degradation rates of 3rd-instar host larvae that were parasitized by Aj treated with *dsRNA* of 15 non-*VID* DUF4803-domain VP genes in clade V. P*_dsGFP_* was used as a control. Three biological replicates were performed. Data are presented as the mean ± SEM. Significance was determined by Welch’s t test (ns, not significant). Representative images of 3rd-instar *nub>EGFP Drosophila* host larvae at 24 h post-parasitization are shown. Wing discs are indicated with white arrowheads. Scale bars: 500 μm.


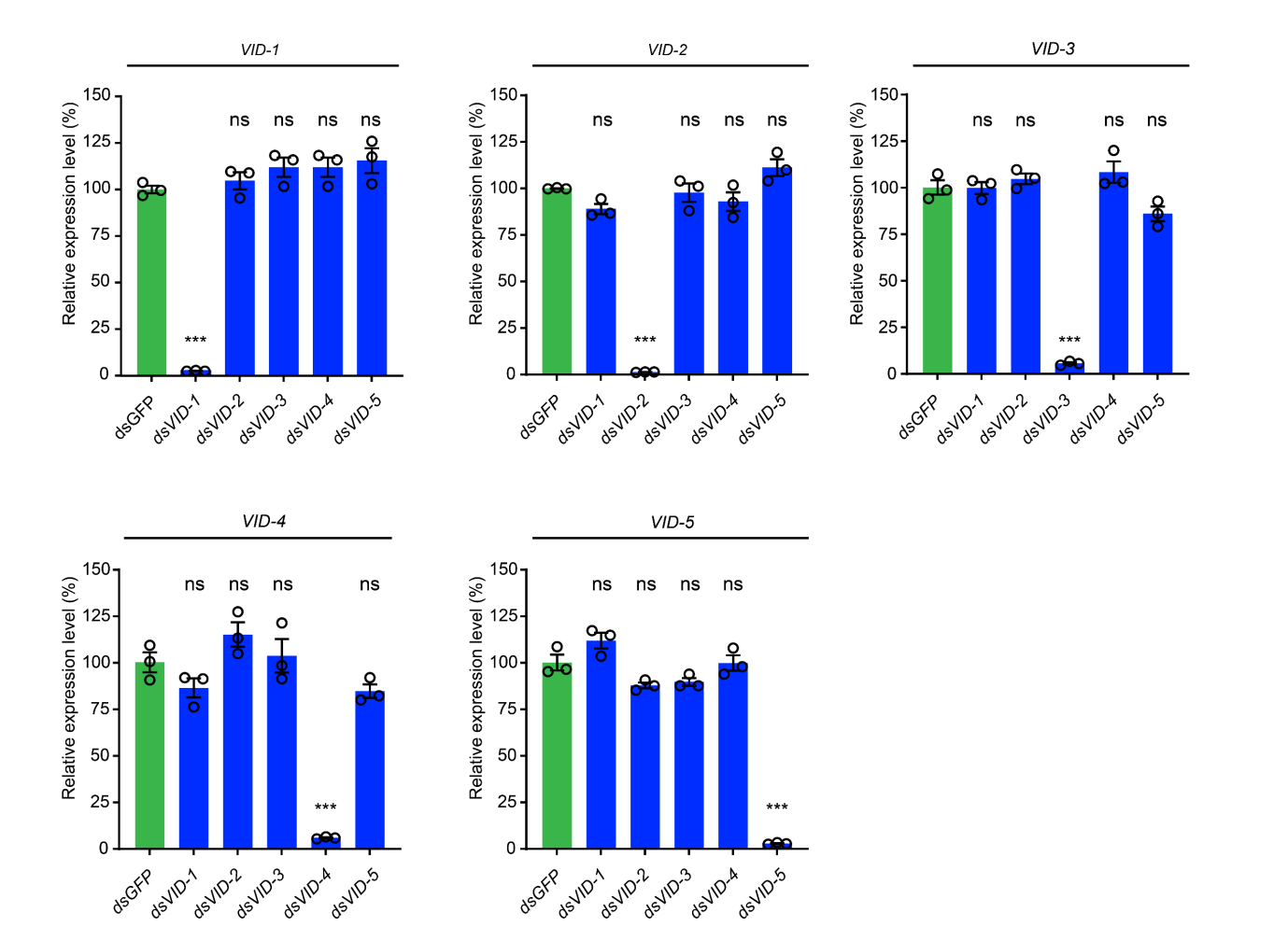
**Figure S16. RNAi effects of five *VID* genes on reducing gene expression.**

Relative expression levels of five *VID* genes (*VID-1*, *VID-2*, *VID-3*, *VID-4*, and *VID-5*) in Aj after *dsRNA* treatment for each specific *VID* gene. The *dsGFP*-treated Aj was used as a control. Three replicates were performed. Data are presented as the mean ± SEM. Statistical analysis was performed by one-way ANOVA with Sidak’s multiple comparisons test (ns, not significant; ***, *p* < 0.001).


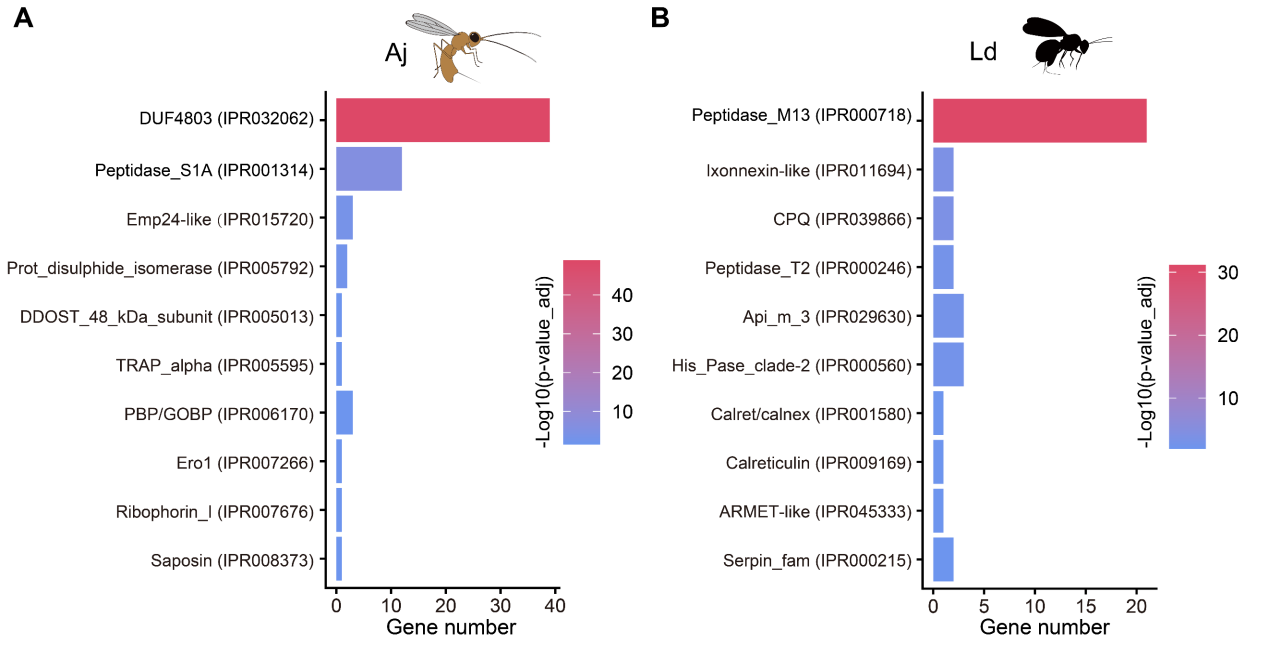
**Figure S17. The enrichment of InterPro families in the VP genes of Ld is completely different from that of Aj.**

The top 10 enriched InterPro families in the VP genes were compared between Aj and Ld. The colour gradient from blue to red represents low to high -log10(p-value) by Bonferroni adjustment via a Hypergeometric test (higher tail). The InterPro families are presented on the y-axis, and the number of InterPro families is presented on the x-axis.


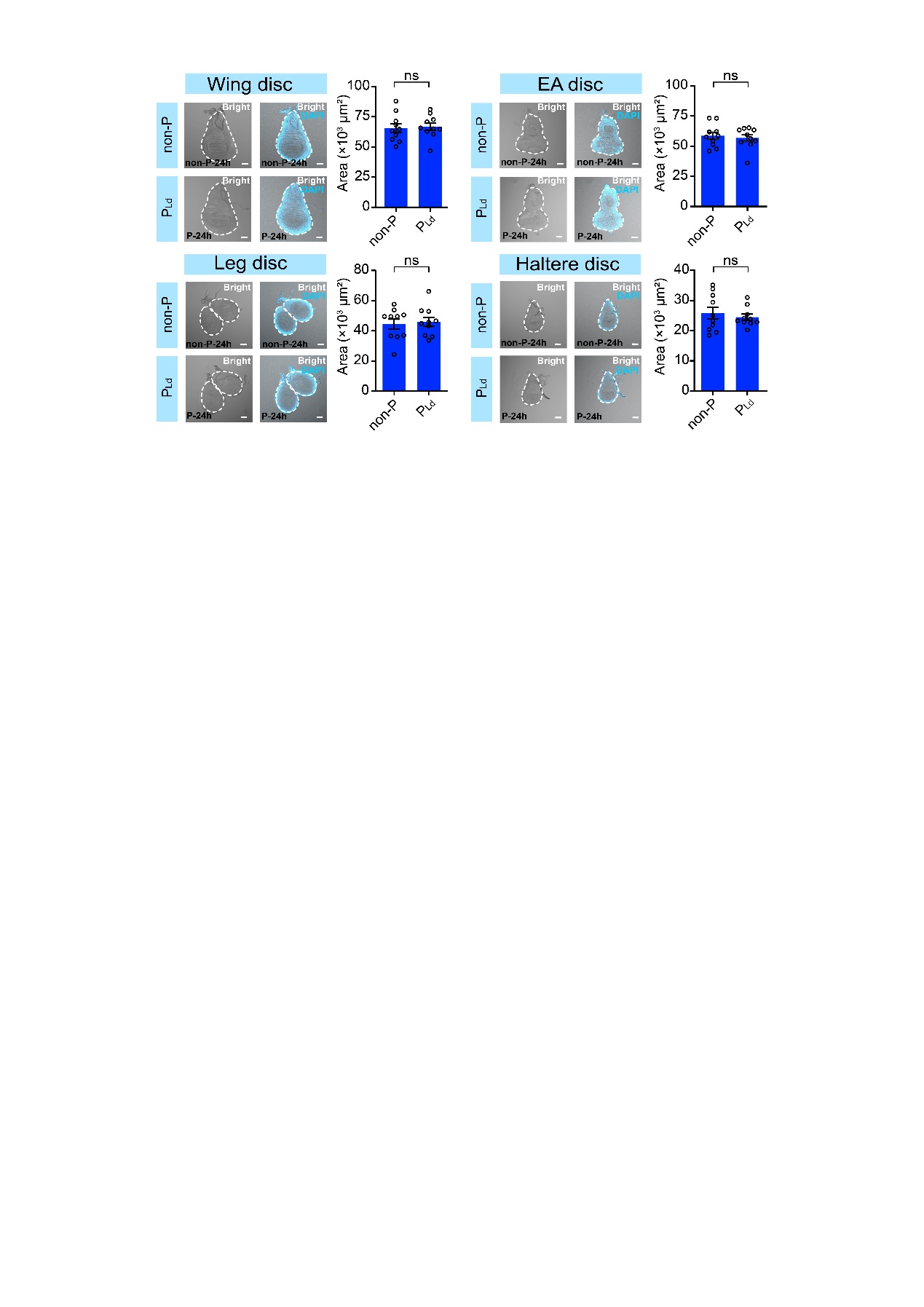
**Figure S18. Ld parasitization does not affect the size of host imaginal discs.**

Representative fluorescence images of wing discs, EA discs, leg discs and haltere discs from non-parasitized (non-P) and Ld-parasitized (P_Ld_) 3rd-instar host larvae at 24 h post-parasitization (P-24h). The bright field image is shown in the Bright channel, while the nuclei of the host imaginal discs are labelled with DAPI (blue). The dash lines mark the outlines of the imaginal discs. At least 10 imaginal discs of each type were analysed for size. The data are presented as the mean ± SEM. Statistical analysis was performed using two-tailed unpaired Student’s t test (ns: not significant). Scale bar: 50 μm.

**Supplementary Table legends**

**Table S1.** The list of upregulated genes in Aj-parasitized wing discs at 6 h post-parasitization.

**Table S2.** The list of upregulated genes in Aj-parasitized EA discs at 6 h post-parasitization.

**Table S3.** Statistical characteristics of the genomic DNA sequencing reads.

**Table S4.** Basic features of the chromosomal-level genome of Aj.

**Table S5.** Aj venom protein (VP) genes.

**Table S6.** Primer sequences used for qRT-PCR.

**Table S7.** RNAi target genes and primers.

**Table S8.** Statistics of RNAseq data in this study.

**Supplementary Movie legends**

**Movie S1:** Movie clip shows a successful oviposition in a second-instar host larva by Ld.

**Movie S2:** Movie clip shows a successful oviposition in a second-instar host larva by Aj.

**Movie S3:** Movie clip shows that Ld induces an intense defensive rolling behaviour in a third-instar host larva (older host), which impeded Ld’s ability to complete oviposition.

**Movie S4:** Movie clip shows a successful oviposition in a third-instar host larva (older host) by Aj.
